# NGR-peptide−drug conjugates with dual targeting properties

**DOI:** 10.1371/journal.pone.0178632

**Published:** 2017-06-02

**Authors:** Kata Nóra Enyedi, Szilárd Tóth, Gergely Szakács, Gábor Mező

**Affiliations:** 1 Eötvös Loránd University, Faculty of Science, Institute of Chemistry, Pázmány P. sétány 1/A, Budapest, Hungary; 2 Institute of Enzymology, Research Center for Natural Sciences, Hungarian Academy of Sciences, Magyar tudósok körútja 2, Budapest, Hungary; 3 Institute of Cancer Research, Medical University Vienna, Borschkegasse 8a, Vienna, Austria; Nanyang Technological University, SINGAPORE

## Abstract

Peptides containing the asparagine-glycine-arginine (NGR) motif are recognized by CD13/aminopeptidase N (APN) receptor isoforms that are selectively overexpressed in tumor neovasculature. Spontaneous decomposition of NGR peptides can result in *iso*Asp derivatives, which are recognized by RGD-binding integrins that are essential for tumor metastasis. Peptides binding to CD13 and RGD-binding integrins provide tumor-homing, which can be exploited for dual targeted delivery of anticancer drugs. We synthesized small cyclic NGR peptide–daunomycin conjugates using NGR peptides of varying stability (c[KNGRE]-NH_2_, Ac-c[CNGRC]-NH_2_ and the thioether bond containing c[CH_2_-CO-NGRC]-NH_2_, c[CH_2_-CO-KNGRC]-NH_2_). The cytotoxic effect of the novel cyclic NGR peptide-Dau conjugates were examined *in vitro* on CD13 positive HT-1080 (human fibrosarcoma) and CD13 negative HT-29 (human colon adenocarcinoma) cell lines. Our results confirm the influence of structure on the antitumor activity and dual acting properties of the conjugates. Attachment of the drug through an enzyme-labile spacer to the *C*-terminus of cyclic NGR peptide resulted in higher antitumor activity on both CD13 positive and negative cells as compared to the branching versions.

## Introduction

**Fig 1:**
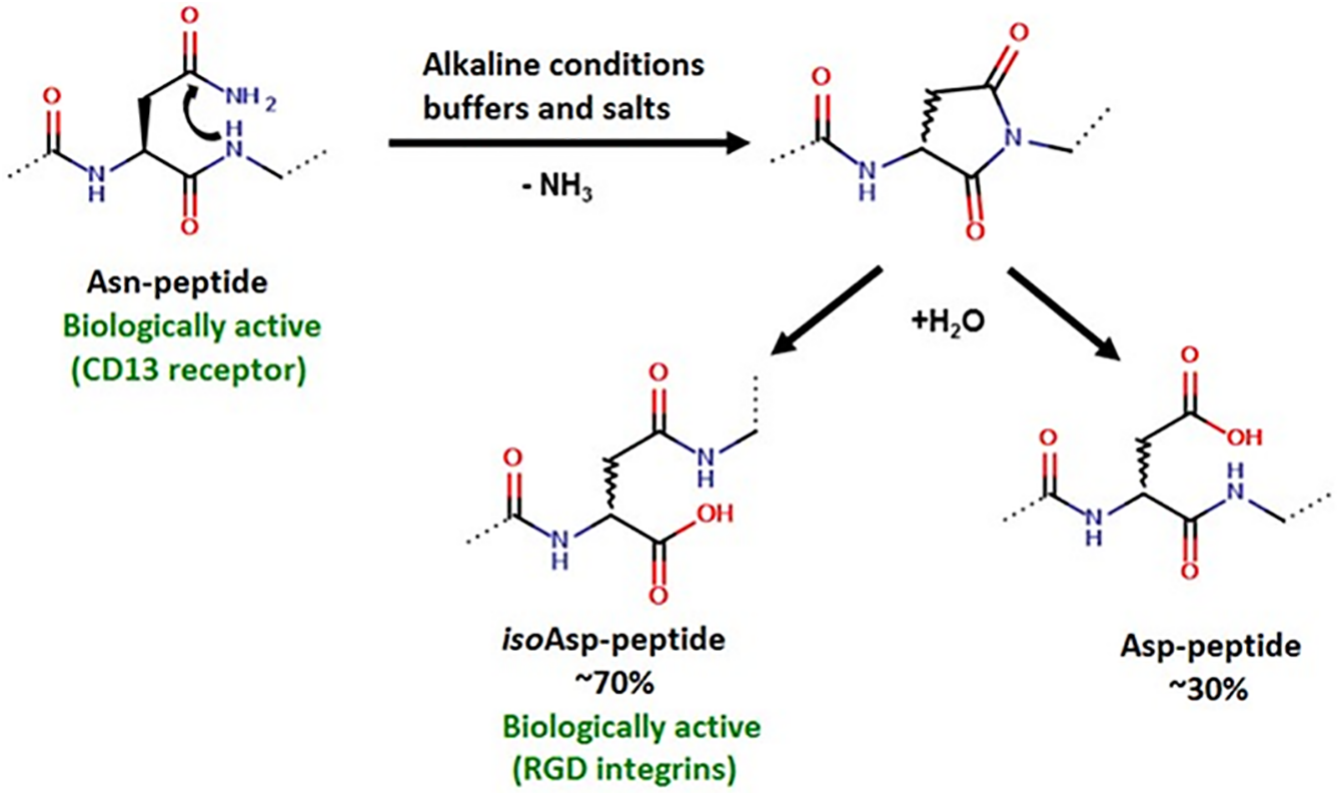
Rearrangement of NGR peptides resulting in dual acting properties.

Most of the currently used anticancer drugs have several shortcomings including poor bioavailability or lack of selectivity, which can result in serious side effects even at therapeutic doses. To reduce the non-specific cytotoxicity of neoplastic agents, targeted drug delivery systems may be used, in which the active compound is conjugated to a homing device. Homing may be mediated by proteins (*e*.*g*. antibodies) or peptides (*e*.*g*. hormone peptides) that recognize tumors with high specificity. The concept of selective drug targeting is based on the high expression of certain cell surface components on tumors or the tumor neovasculature [1]. It is well known that solid tumors recruit new blood vessels to support tumor growth, therefore tumor angiogenic vessels might also be used clinically to develop better targeted therapy [2]. One of the cancer specific receptors is aminopeptidase N (APN or CD13). APN is a membrane-bound metalloprotease, which can be found not only in tumors and angiogenic blood vessels, but it is also expressed in various normal cell types (*e*.*g*., liver, prostate, kidney, renal). However, experiments with APN antibodies have shown that different APN isoforms are expressed in cancer associated endothelial cells and in healthy tissues and blood vessels [3]. Since APN plays a key role in angiogenesis, cell invasion and cell proliferation, it is considered an ideal target for selective anticancer drug delivery systems [4,5]. The relevance of APN ligands (NGR (Asn-Gly-Arg) sequence containing peptides) in tumor targeting was discovered in *in vitro* screens using phage-display libraries set up to identify non-RGD (-Arg-Gly-Asp-) integrin binding motives [6–8]. Later, it was shown that NGR peptides bind to a CD13 isoform, which is expressed in tumor blood vessels where it functions as a vascular receptor for the NGR motif containing extracellular matrix (ECM) proteins [3]. The NGR motif is prone to non-enzymatic deamidation through succinimide ring formation followed by hydrolysis, resulting in aspartyl and isoaspartyl (DGR and *iso*DGR) derivatives. Studies have shown that the formed isoaspartyl (*iso*DGR) derivative is the ligand of RGD-integrins, which are essential for tumor angiogenesis, cell invasion and metastasis [9]. Through NGR-to-*iso*DGR rearrangement NGR-peptides are able to bind to both CD13 and RGD binding integrin, which is why these peptides are considered ideal candidates for selective, dual acting, tumor targeting ligands (Fig 1).

Several NGR-containing peptide sequences have been identified and optimized through phage-display libraries. Nevertheless, only a few NGR peptides have been exploited for their specific dual tumor and tumor vessel homing ability [10,11]. There are examples for both linear and cyclic NGR peptides, but as a general rule, small cyclic NGR peptides are superior compared to their linear forms in terms of selectivity [12–14]. The increased efficiency due to cyclization is a common phenomenon and many examples are known, where conformational constraining led to improved affinity [15]. At present, the two widely used effective and tumor selective cyclic NGR peptides are [CNGRC] [10] and [KNGRE]-NH_2_ [11].

The disulfide bridge containing [CNGRC] was applied for targeted delivery of various chemotherapeutic drugs (*e*.*g*. Pt-complex, doxorubicin) [16,17], viral particles [18], proapoptotic peptides [19], radioisotopes [20], cytokines [21–25] and siRNA [26]. Anthracyclines can also inhibit cell growth through antiangiogenic pathways [27,28]. Therefore, anthracycline–NGR peptide-based conjugates are excellent candidates for vascular endothelium targeted tumor therapy. Dox-[CNGRG] conjugate showed antitumor effect both *in vitro* and *in vivo*, but no significant selectivity between CD13 positive and negative tumor cells/tissues was observed [17,29]. However, this version of cyclic NGR was formed by using a labile disulfide bridge between the flanking cysteines, which may complicate the synthesis of derivatives and conjugates.

As a more stable alternative of [CNGRC] intended for wider applications, Negussie *et al*. designed the “head-to-side chain” amide bond cyclized [KNGRE]-NH_2_ peptide [12]. Although this peptide is identical in terms of cycle size (17 atoms), the NGR motif showed increased stability. Because of this characteristic, [KNGRE]-NH_2_ has been studied for tumor targeting applications, for delivery of liposomes [12], fluorescent dyes [12,30] and radioisotopes for PET imaging [31].

In our previous study we developed small cyclic NGR peptides containing a thioether bond and compared their chemostability [32]. Based on these results, in the present study our aim was to (i) design and synthesize novel cyclic NGR peptide-drug conjugates; (ii) follow and characterize the rate of succinimide formation and the rearrangement of the conjugates; (iii) measure their cytotoxic effect *in vitro*; and (iv) understand the structure-biological activity relationship of the conjugates, in hope of creating effective NGR-based drug delivery systems as chemotherapeutics for tumor targeting.

## Materials and methods

All reagents and solvents (anhydrous) were obtained from VWR International Kft. (Debrecen, Hungary). Fmoc-Rink Amide MBHA resin, *N*,*N′*-diisopropylcarbodiimide (DIC), 1,8-diazabicyclo[5.4.0]undec-7-ene (DBU), 1-hydroxybenzotriazole hydrate (HOBt), and ninhydrin were purchased from Sigma-Aldrich Kft (Budapest, Hungary). Fmoc-Rink Amide 2CT resin and all amino acid derivatives used in this study were purchased either from Merck KGaA (Darmstadt, Germany) or Iris Biotech GmbH (Marktredwitz, Germany).

### Preparation of isopropylidene protected aminooxyacetic acid

Aminooxyacetic acid (0.7 g (7.7 mmol) (*O*-carboxymethyl)hydroxylamine)) was dissolved in 25 mL technical grade acetone and was allowed to react for 30 minutes, followed by evaporation of the excess of acetone. The remaining oily product was solidified with diethyl ether. After removal of ether the white crystals were dried *in vacuo* overnight. The product (0.89 g, yield: 94%) was identified by ESI-MS and NMR (S1 Fig).

### Solid phase synthesis of branched precursor NGR/DGR peptides

The branched NGR and DGR peptides were synthesized manually on Fmoc-Rink Amide MBHA resin (0.71 mmol/g loading capacity) using standard protecting schemes except for the synthesis of the KDGRE derivative. In this case the highly acid-labile Fmoc-Rink Amide 2CT resin (0.67 mmol/g loading capacity) and *O*-2-PhiPr (2-phenylisopropyl ester) side chain protecting group for Glu were used. The applied lysine derivatives were Fmoc-Lys(Dde)-OH, Dde-Lys(Fmoc)-OH and Boc-Lys(Fmoc)-OH depending on their position in the peptide sequences. The protocol of the modified Fmoc/^*t*^Bu strategy was as follows: (i) DMF washing (4 x 0.5 min), (ii) Fmoc deprotection with 2% DBU, 2% piperidine, 0.1 M HOBt in DMF (2 x 2 min, 2 x 5 min, 2 x 10 min), (iii) DMF washing (10 x 0.5 min), (iv) coupling of Fmoc-protected amino acid derivative: DIC: HOBt in DMF (3 equiv each) (1 x 60 min), (v) DMF washing (3 x 0.5 min), (vi) DCM washing (2 x 0.5 min), (vii) ninhydrin assay. The cleavage of Dde protecting group to form the branches was carried out in 2% hydrazine in DMF for 6 x 3 min. The standard DIC/HOBt coupling method was used for the incorporation of isopropylidene protected aminooxyacetic acid to the *N*-terminus of GFLG spacer sequence in all cases.

The peptide-loaded resins were stirred for 3 h at RT in 10 mL of cleavage mixture (95% TFA, 2.5% triisopropylsilane (TIS), 2.5% H_2_O) for the removal of peptides from the resins and deprotection of the side chains, except for the KDGRE control peptide. The crude products were precipitated with ice cold diethyl ether and centrifuged for 4 min at 4000 rpm. After washing three times with diethyl ether, the crude products were dissolved in water and the solution was purified by RP-HPLC and lyophilized. The cleavage of precursor for control peptide with KDGRE sequence from the resin and removal of the *O*-2-PhiPr side chain protecting group occurred in 10 mL of 2% TFA, 2%TIS/DCM for 2 h at RT. After separation from the resin beads the cleavage mixtures were evaporated and the remaining semi-protected product was precipitated with d.i. water, collected by centrifugation (4 min at 4000 rpm), dissolved in acetonitrile-water and lyophilized.

### Cyclization of precursor peptides

The thioether linkage and disulfide bridge were formed in 0.1 M TRIS buffer (pH 8.1) at a peptide concentration of 1 mg/mL for 1 h. The mixtures were then adjusted to pH 7 with TFA and purified by RP-HPLC and lyophilized.

In the case of the amide bond formation salt exchange was performed using 10 equiv of pyridinium hydrochloride in 5 mL MeOH. After 20 min MeOH was evaporated and the remaining oily products were dissolved in DMF at a concentration of 1 mg/mL and BOP, HOBt and DIPEA (3:3:6 equiv) were added to the solution. The mixtures were stirred for overnight and then the DMF was evaporated. The oily products were dissolved in acetonitrile-water and purified by RP-HPLC and the collected fractions were lyophilized. The remaining side chain protecting groups were cleaved according to the standard protocols before the drug conjugation procedure, as described above.

### Conjugation of daunomycin to cyclic NGR/DGR peptides

Prior to the conjugation of daunomycin (Dau) the isopropylidene protecting group was removed with 1 M solution of methoxyamine in 0.2 M NH_4_OAc solution (pH 5). The peptides were dissolved in 1 mg/mL concentration, stirred at RT for one hour and were purified by RP-HPLC. The solvent was evaporated from the collected fractions and the conjugations were carried out in solution at once. The cyclic, deprotected peptides were dissolved in 0.2 M NH_4_OAc solution (pH 5.0) at a 10 mg/mL concentration, and 1.5 equiv of Dau was added. The mixtures were stirred overnight then they were purified by RP-HPLC and lyophilized. The resulting conjugates were characterized by electrospray ionization mass spectroscopy (ESI-MS).

### Reverse phase high performance liquid chromatography (RP-HPLC)

The crude peptides and conjugates were purified on a KNAUER 2501 HPLC system (KNAUER, Bad Homburg, Germany) using a semi-preparative Phenomenex Jupiter Proteo C18 column (250 mm x 10.0 mm) with 10 μm silica (90 Å pore size) (Torrance, CA). Linear gradient elution (0 min 5% B; 65 min 90% B) with eluent A (0.1% TFA in water) and eluent B (0.1% TFA in MeCN-H_2_O (80:20, v/v)) was used at a flow rate of 4 mL/min. Peaks were detected at 220 nm. Analytical RP-HPLC was performed on the same instrument using a Phenomenex Aeris Peptide XB-C18 column (250 mm x 4.6 mm) with 3.6 μm silica as a stationary phase. Linear gradient elution (0 min 0% B; 5 min 0% B; 50 min 90% B) using the same eluents was applied at a flow rate of 1 mL/min. Peaks were detected at 220 nm.

### Electrospray ionization mass spectrometry (ESI-MS)

ESI-MS analyses were carried out on an Esquire 3000+ ion trap mass spectrometer (Bruker Daltonics, Bremen, Germany). Spectra were acquired in the 50–2000 *m/z* range. Samples were dissolved in a mixture of 20% acetonitrile and 80% water.

### Chemostability measurements

For the stability studies the conjugates were dissolved in DMSO (2% of the final volume), then 10% FBS (fetal bovine serum) containing complete cell culture medium (DMEM CM) was added up to the 1 mg/mL final concentration of the conjugates followed by incubation at 37°C. Samples were taken from the mixtures at 0 h, 6 h and after 72 h, respectively, and components were separated with Amicon Ultra Centrifugal Filters (cut off 10K, Millipore) in two steps: (i) The membranes were preconditioned with eluent A (used for HPLC), then samples diluted with eluent A were added (final volume was 0.5 mL) and centrifuged at 13000 rpm for 15 min and was repeated three times with each sample. Due to daunomycin adsorption ability, only the small molecules of the cell medium eluted at this stage. (ii) In the next step, eluent B (used for HPLC) was added (final volume was 0.5 mL) and centrifuged at 13000 rpm for 15 min, followed by lyophilisation to concentrate the samples.

### Cell lines and culture conditions

The HT-1080 fibrosarcoma cell line was a kind gift from Dr. József Tóvári (National Institute of Oncology, Hungary), the HT-29 human colon cancer cell line was obtained from DCTD Tumor Repository (NCI at Frederick, Maryland). HT-1080 and HT-29 cells were maintained in DMEM and RPMI (Sigma Aldrich), respectively, supplemented with 10% FBS, 5 mmol/L glutamine, and 50 units/mL penicillin and streptomycin (Life Technologies). All cell lines were cultivated at 37°C, 5% CO_2_. Cells were periodically tested and resulted negative for mycoplasma contamination with the MycoAlert™ mycoplasma detection kit (Lonza, Hungary).

### Detection of CD13 receptors on tumor cells by flow cytometry

The surface expression of CD13 was determined with a FITC-conjugated OKM13 monoclonal murine antibody (Ortho Diagnostic systems). Briefly, 250,000 cells (in suspension) were stained with 5 μL of the FITC-conjugated OKM13 for 30 min at 37°C. After the incubation, cells were washed with PBS and 1 μL PI solution was added to distinguish live/dead cells. Samples were measured and analyzed with an Attune® Acoustic Focusing Cytometer (Life Technologies).

### *In vitro* cytostatic effect and cytotoxicity

Cells were seeded in 5000 cells/well density in 100 μL medium, and were incubated overnight. The following day 100 μL of serially diluted drugs were added to the cells. In the case of the cytostatic effect measurements, drug containing medium was gently removed from the plates after 6h incubation, fresh medium was added to each wells, and the plates were further incubated for additional 66 h (72 h in total). In the case of cytotoxicity measurements, the drug containing medium was on the cells for the full period of the 72 h assay. At 72 h, supernatant was removed from the cells, and viability was assessed by the PrestoBlue® reagent (Life Technologies), which was diluted in PBS to reach the concentration given in the manufacturer’s instruction. Curves were fitted by GraphPad Prism 5 software using the sigmoidal dose–response model (https://www.graphpad.com/scientific-software/prism/). The conjugates were serially diluted, 9 concentrations were measured in triplicates. The tests were carried out 3–4 times; IC_50_ values were averaged; statistical significance was calculated using unpaired *t*-tests.

### Lysosomal degradation

Rat liver lysosomal homogenate was prepared as described previously [33]. Protein concentration was determined with bicinchoninic acid (Pierce BCA protein assay) according to the manufacturer’s protocol (ThermoFisher Scientific, Rockford, IL, USA) and it was 17.4 μg/μL. Degradation of the bioconjugates in the rat liver lysosomal homogenate was determined as follows: i) bioconjugates were dissolved in d.i. water at a concentration of 4 μg/μL, ii) the solutes were further diluted to a concentration of 0.2 μg/μL with 0.2 M NaOAc (pH 5.0), iii) the rat liver lysosomal homogenate was diluted with 0.2 M NaOAc (pH 5.0) to a concentration of 3.48 μg/μL, iv) the homogenate was added to the conjugates (conjugate/lysosomal homogenate ratio = 1:1, w/w), v) the reaction mixtures were incubated at 37°C and aliquots of 13 μL were taken at 5 min, 6 h and 72 h, vi) the reactions were quenched by adding 2 μL of formic acid, followed by LC-MS analysis (Bruker Esquire 3000+; Jasco PU-2085plus system; Supelco Ascentis C18 coloum, 3 μm, 2,1 x 150 mm, 100 Å).

### Cellular uptake

HT-1080 and HT-29 cells were seeded at a 250,000 cells/well density, and were incubated overnight in their culturing media. The following day daunomycin conjugates were dissolved in culturing media (FBS supplemented), and added to the cells immediately at 10 μM final concentration. After 6 h incubation at 37°C the supernatant was removed, cells were washed with PBS and trypsinized with 0.1% trypsin (Gibco® by Life Technologies) for 10 minutes. Trypsinization was terminated with FBS containing medium, then cells were washed and suspended in serum free medium. For live/dead distinction we used Zombie Violet reagent (BioLegend, San Diego, CA). Samples were measured and analyzed with an Attune® Acoustic Focusing Cytometer (ThermoFisher Scientific).

## Results and discussion

**Fig 2:**
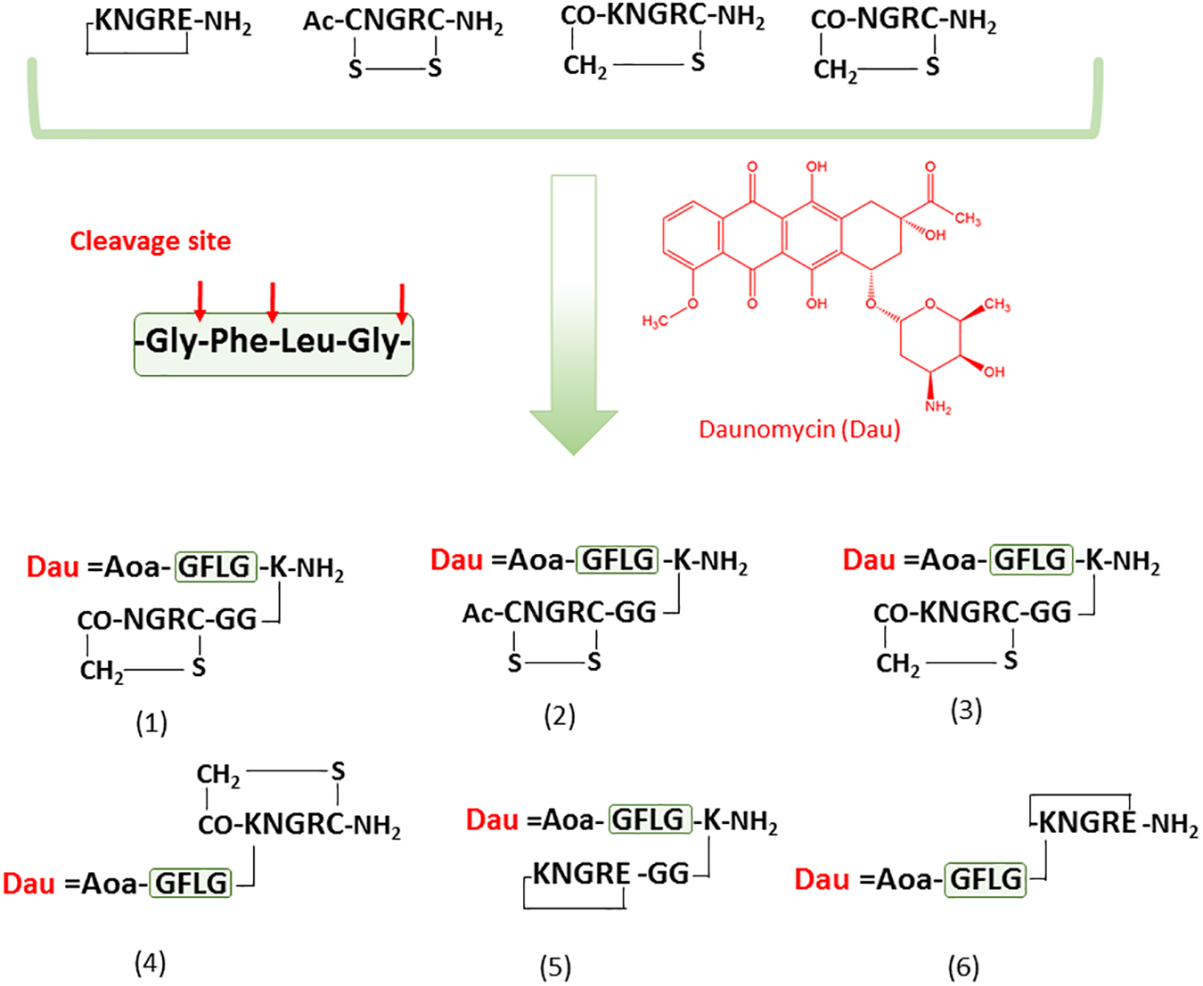
Structure of the cyclic NGR peptides and their Dau conjugates.

Cyclic NGR peptides are promising candidates as targeting moieties for drug delivery to CD13-positive tumor cells and the tumor neovasculature. Up to now the most widely used NGR peptide conjugates have been based on the disulfide bridge containing cyclic peptide [CNGRC]. The cargo (including tumor necrosis factor (TNF), platina complexes, etc.) is usually attached to the *C*-terminus of the peptide [17,24]. Recently, an amide bond containing cyclic NGR peptide ([KNGRE]-NH_2_) with higher resilience against deamidation has been described, in which cargos such as fluorescent labels or chelated ^68^Ga for PET were attached to the lysine side chain [12,31]. Several cyclic NGR peptides with thioether linkage were developed in our laboratory [32]. Compared to the amide bond or the disulfide bridge, the enzyme stability of the thioether linkage is significantly higher and it can be formed more easily [34]. Conversely, it was suggested that the conjugates with thioether linkage have a lower chemostability to deamidation compared to the previously mentioned disulfide and amide bond containing versions [32]. However, deamidation is highly influenced by the structure of cyclic peptides. The extremely labile cyclic peptides produce isoaspartyl derivatives, which may be useful in a dual targeting approach in which the attached drugs are delivered to both CD13 receptor and the RGD-binding integrins. Based on our previous study we selected four cyclic NGR peptides for drug targeting: ([KNGRE]-NH_2_, Ac-[CNGRC]-NH_2_, [CH_2_-CO-NGRC]-NH_2_, [CH_2_-CO-KNGRC]-NH_2_) representing stable, moderately and two highly labile thioether bond-containing compounds, respectively [32]. The cyclic NGR peptides were attached to daunomycin (Dau) with good yield *via* oxime bond formation [35]. In contrast to ester linked doxorubicin [17,29] the oxime linkage results in a conjugate which is stable in human plasma. Although the free drug is not released from the oxime linkage [33], conjugates show higher tumor growth inhibition and antiangiogenic effect *in vivo* compared to the free drug used at MTD [36]. In addition, this approach is a cost effective and useful method for searching appropriate homing peptides following the development of other drug–peptide conjugates [37]. Therefore, Dau was linked to the cyclic NGR peptides *via* oxime linkage using aminooxyacetic acid (Aoa) attached to a Cathepsin B labile GFLG tetrapeptide spacer to ensure efficient drug release. The lysosomal enzyme Cathepsin B is highly upregulated in various cancer cells, which results in the selective release of the free drug or its active metabolite in tumors (Dau = Aoa-Gly-OH as the smallest metabolite in these cases). The Dau = Aoa-GFLG part of the molecule was connected directly to the side chain of Lys in the cycle or to the *C*-terminus of the cyclic peptide through an additional Lys. The *C*-terminal elongated variants were extended with two glycine residues to prevent incidental structure-based reduction of efficiency. Six novel NGR peptide–Dau conjugates were developed (Fig 2) and their stability, lysosomal degradation, cellular uptake as well as cytostatic/cytotoxic effects were measured. In addition, the conjugates in which Asn was replaced by Asp were also prepared as controls to allow the identification of the deamidated compounds in stability studies.

### Synthesis of cyclic NGR peptide-Dau conjugates

Linear precursor peptides were prepared on Fmoc-Rink Amide MBHA or Fmoc-Rink Amide 2CT resins using Fmoc/^t^Bu strategy. Through the syntheses a modified Fmoc cleavage mixture and protocol was used to hinder undesirable succinimide ring formation.

**Fig 3:**
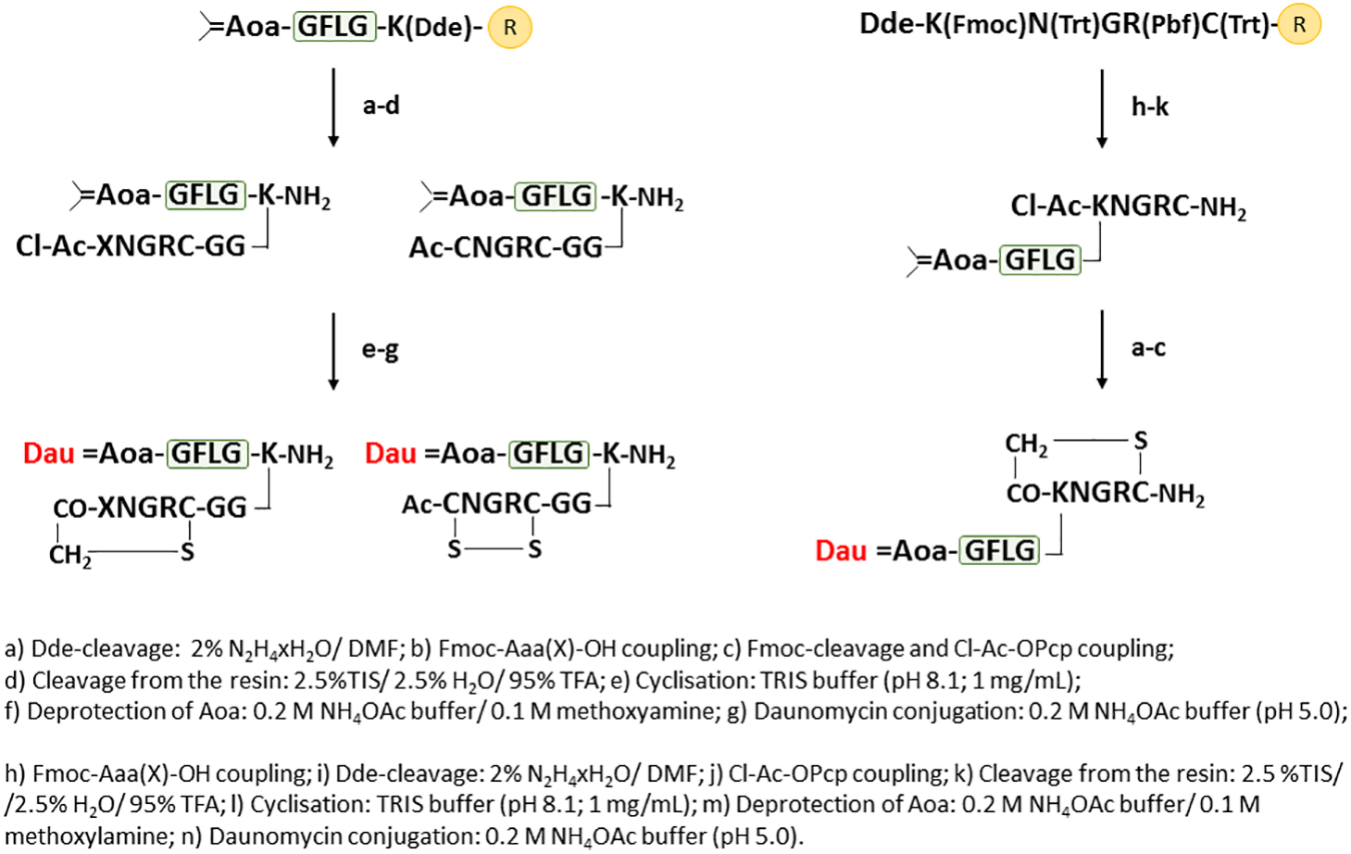
Synthesis of Dau-NGR peptide conjugates with disulfide bridge or thioether bond in the cycle.

In cases of conjugates **1, 2, 3, 5**, the enzyme labile spacer with isopropylidene protected amoniooxyacetyl moiety was built up using a Fmoc-Lys(Dde)-OH, as the *C*-terminal amino acid derivative (Fig 3). The isopropylidene protected aminooxy moiety (S1 Fig) is stable under the conditions applied for the synthesis and cleavage from the resin. The second step was the removal of the Dde as lysine side chain protecting group, followed by coupling two glycine residues and the specified NGR moieties. Peptide **5** contains Boc-Lys(Fmoc) in the KNGRE sequence, which allowed the selective “head-to-side chain” cyclization after the cleavage of the semi-protected peptide from the resin.

**Fig 4:**
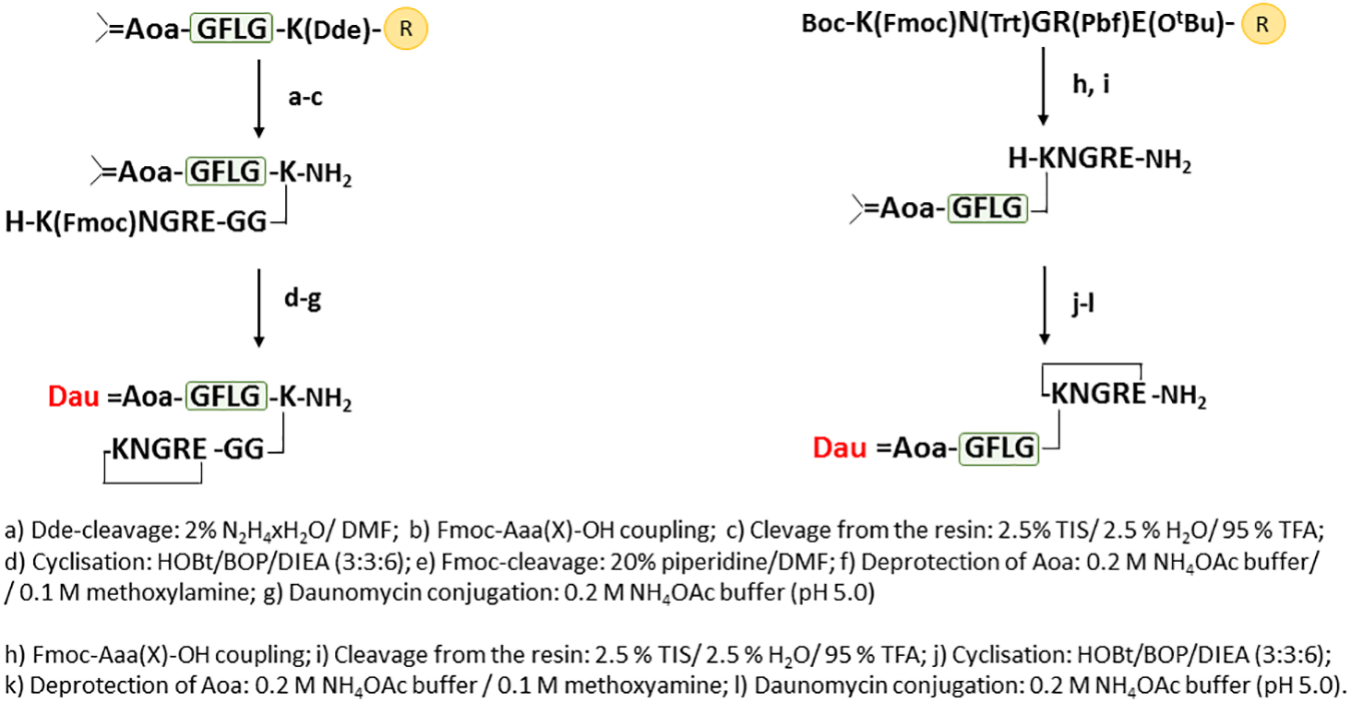
Synthesis of Dau-NGR peptide conjugates with amide bond in the cycle.

Synthesis of branched conjugates **4** and **6** was performed in a reverse order: first the NGR sequences were prepared with incorporation of Dde-Lys(Fmoc)-OH (conjugate **4**) and Boc-Lys(Fmoc)-OH (conjugate **6**) (Fig 4). Next, the Fmoc side chain protecting group was removed, which allowed the synthesis of isopropylidene protected Aoa-GFLG spacer on the ε-amino group of Lys.

For the development of cyclic NGR peptides *via* thioether linkage (conjugate **1**, **3** and **4**) an additional chloroacetyl group was incorporated to the *N*-terminus of the NGR sequence at the last step using chloroacetic acid pentachlorophenyl ester.

**Fig 5:**
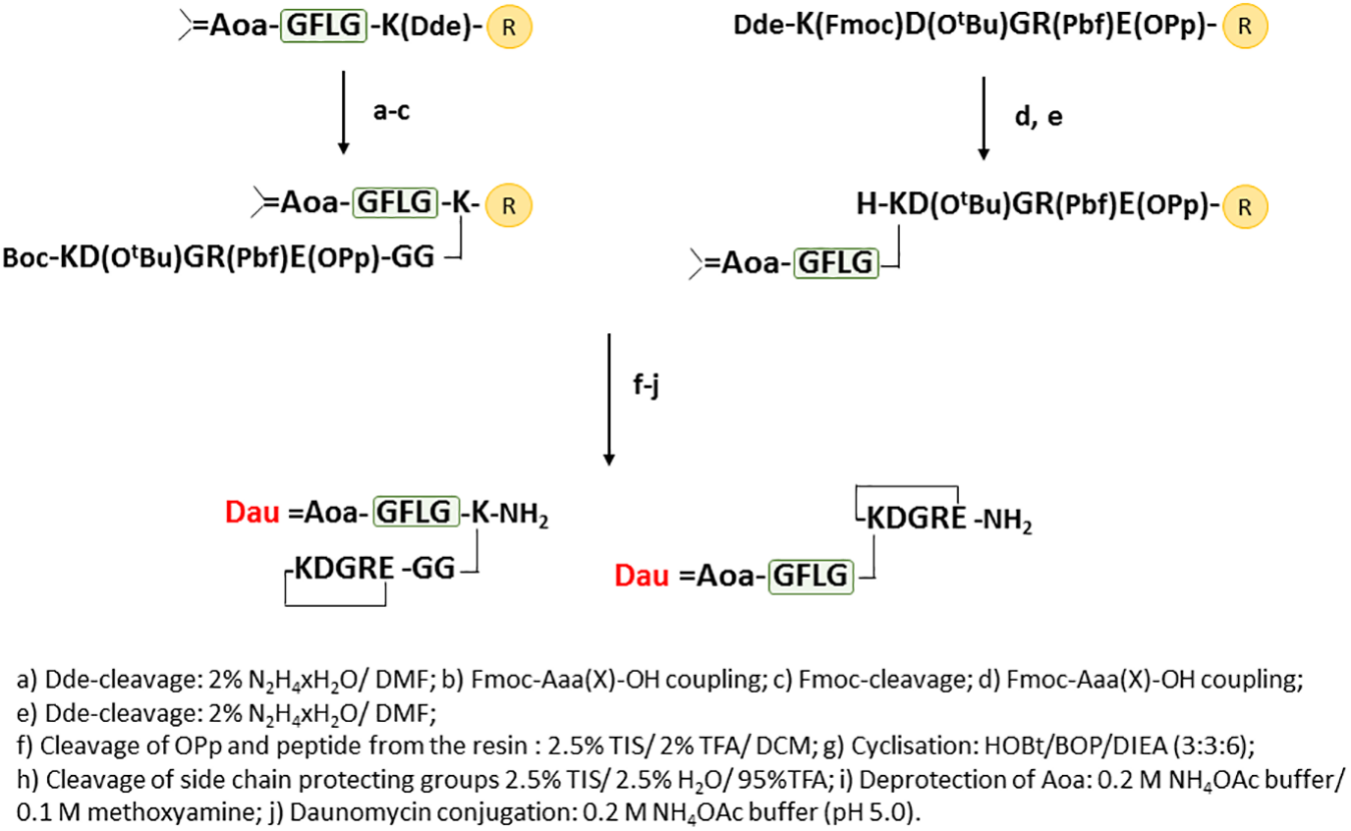
Synthesis of the control Dau-DGR peptide conjugates with amide bond in the cycle.

The synthetic protocol of the control DGR derivatives of **1**, **2**, **3**, **4** was the same for the NGR peptides (Fig 3), except that Fmoc-Asp(O^t^Bu)-OH was used instead of Fmoc-Asn(Trt)-OH. Because DGR derivatives of compound **5** and **6** contain both Asp and Glu, a different synthetic route was needed (Fig 5). For this purpose, the highly acid sensitive Fmoc-Rink Amide 2CT resin and the highly acid sensitive Fmoc-Glu(OPp)-OH amino acid derivative were applied for selective removal that allow “head-to-side chain” cyclization. In each case the cyclization was carried out in TRIS buffer (pH 8.1) for thioether linkage (for 3 h) and disulfide bond formation (air oxidation for overnight) or in DMF in the presence of BOP/HOBt/DIPEA for amide bond formation. Removal of isopropylidene protection from aminooxyacetyl moiety and Dau conjugation was achieved in an NH_4_OAc solution (pH 5.0), as shown in Figs 3–5.

**Fig 6:**
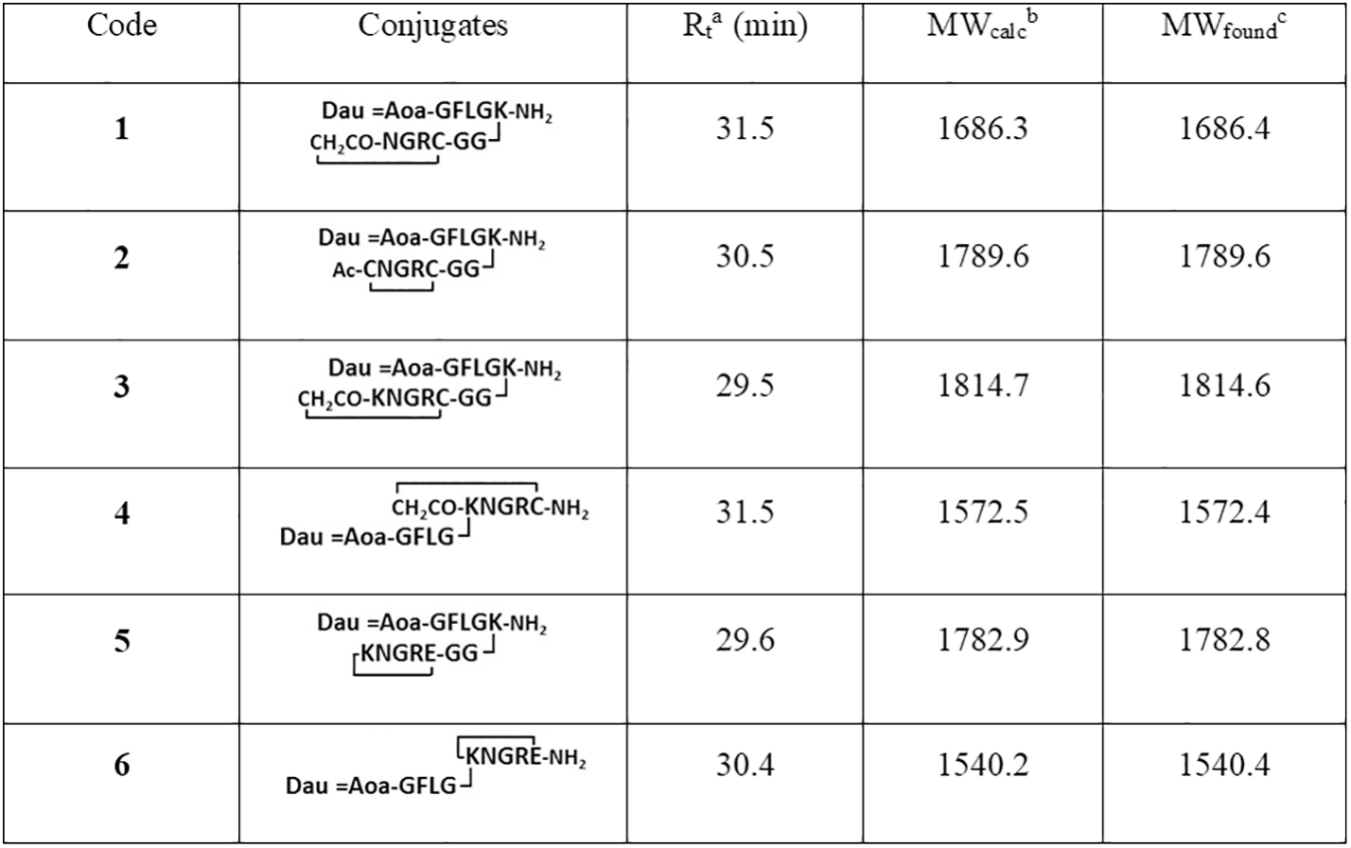
Characteristics of cyclic NGR peptide–daunomycin conjugates. ^a^HPLC column: Phenomenex AERIS Peptide 3,6 μm XB-C18 (250 x 4,6 mm); eluents 0.1% TFA/ water (A) and 0.1% TFA/CH3CN−water (80:20 v/v) (B); gradient 0 min 0% B, 5 min 0% B, 50 min 90% B; flow rate 1 mL/min; detection: 220 nm. ^b^Average molecular weight. ^c^ESI-MS: Bruker Daltonics Esquire 3000+ ion trap mass spectrometer.

The analytical characteristics of the cyclic NGR peptide-drug conjugates are summarized in Fig 6. (The HPLC chromatograms and the mass spectra of the purified cyclic peptides are presented in Supporting Information (S2–S7 Figs). Purity of the cyclic peptides was over 95% in all cases.

### Chemostability measurements of cyclic NGR peptide-Dau conjugates

**Fig 7:**
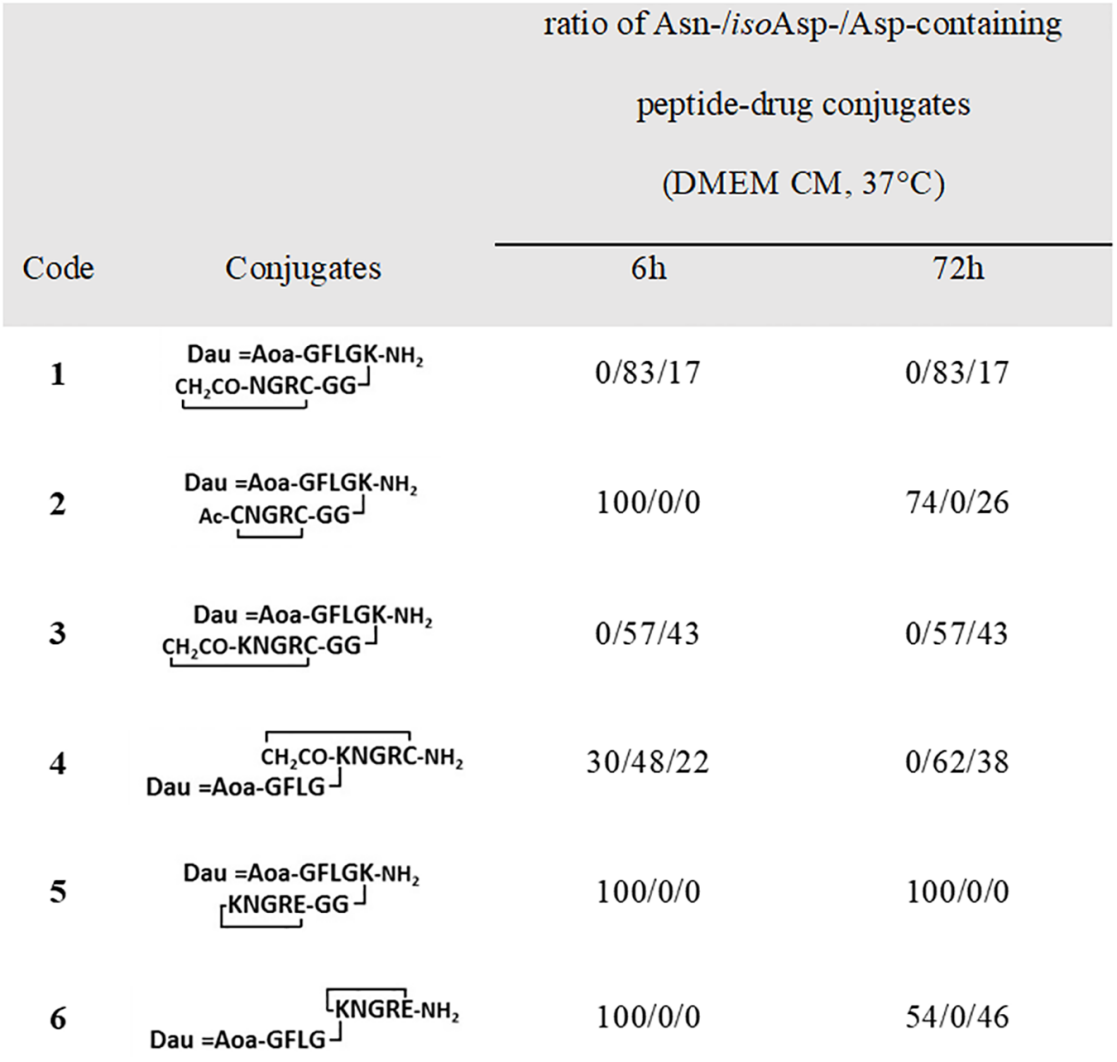
Chemostability of cyclic NGR peptide–daunomycin conjugates.

The stability of cyclic NGR peptide-drug conjugates was determined in DMEM cell culture medium containing 10% FBS (fetal bovine serum), under conditions mimicking *in vitro* cytotoxicity experiments (incubation at 37°C). Samples for dialysis were taken at 6 h and 72h. Except deamidation no other decomposition of the compounds was observed during the incubation time. The deamidation rate was followed by analytical RP-HPLC and was calculated from the area under the curve (AUC). The compounds were identified by both mass spectrometric analyses and by the HPLC retention time of the reference DGR peptide conjugates. The results of chemostability measurements are summarized in Fig 7, chromatograms are presented in the Supporting Information (S2–S7 Figs).

The results showed that the stability characteristics of the conjugates were similar to those observed for non-conjugated small cyclic NGR peptide derivatives in an earlier study [32]. Decomposition of the thioether bond containing cyclic peptide conjugates (**1**, **3** and **4**) was faster as compared to the disulfide (**2)** and amide bond (**6** and **5**) containing conjugates. Interestingly, the amide and thioether bond containing conjugates showed different stability depending on the conjugation site. Lower stability was observed in case of the amide bond containing conjugate **6,** in which the drug-spacer element was attached directly to the side chain of Lys, compared to the conjugate **5**. In contrast, conjugates containing a thioether linkage in the cycle showed faster deamidation when the drug-spacer moiety was attached to the *C*-terminus of the cyclic peptide. The most stable compound was conjugate **5** that did not decompose at all during the time of measurement. Furthermore, it seems that during the time frame of the experiment, decomposition of **2** and **6** resulted only in Asp derivatives without any *iso*Asp formation under the examined conditions. This observation contradicts the results of the previous study on unconjugated cyclic NGR peptides [32], which suggested that the direction of the decomposition is influenced by the drug-spacer moiety. These results suggest that the decomposition of the conjugates cannot be predicted from results obtained with the free cyclic peptide. The *iso*Asp/Asp ratio after deamidation of conjugates **1**, **3** and **4** in DMEM was about the same (~ 2:1) as observed with the free peptides. In conclusion, the stability data suggest that the thioether bond containing cyclic NGR peptides might be good candidates for dual targeting to both CD13 and RGD integrin receptors.

### Cytostatic/cytotoxic effects of NGR-peptide-Dau conjugates

**Fig 8:**
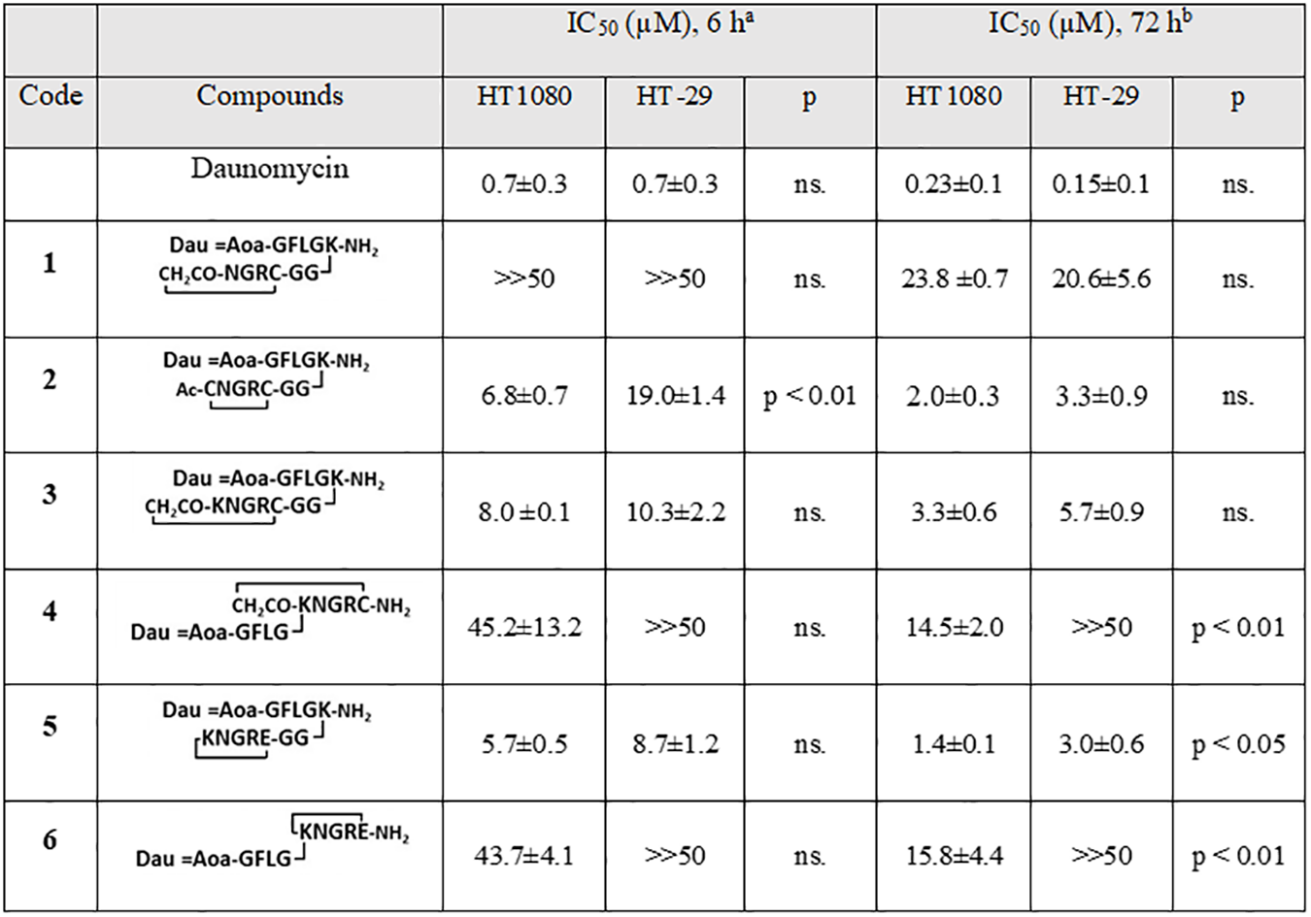
Cytostatic/cytotoxic effect of cyclic NGR peptide–daunomycin conjugates. ^a^Tests were carried out 4 times; ^b^tests were carried out 3 times; IC_50_ values were averaged; statistical significance was calculated using unpaired t-tests.

The cytostatic/cytotoxic effect of the cyclic NGR peptide-Dau conjugates was examined *in vitro* on CD13-positive HT-1080 human fibrosarcoma cells and on CD13-negative HT-29 human colon adenocarcinoma cells. High expression of CD13 in HT-1080 cells was confirmed by FACS, using the CD13-specific OKM13 antibody. In contrast, HT-29 cells do not express any CD13 (S8 Fig). Since both type of cells express RGD integrin receptors [38], but only HT-1080 cells express CD13 receptors, a comparative study of these cells allows the analysis of structure-activity relationship and selectivity. Since several integrin receptors (*e*.*g*. α_v_β_3_, α_v_β_5_, α_v_β_6_, α_5_β_1_) can recognize *iso*DGR peptides [39,40], expression of any specific integrin receptors was not investigated. We compared the toxicity of the conjugates and the free drug molecules. It is important to note that the free drug enters the cell *via* diffusion, while the conjugates are taken up by a receptor-mediated pathway, which is followed by the intracellular release of the drug molecule or its active metabolite. Therefore, the *in vitro* toxicity of oxime-linked drug conjugates is expected to be inferior to that of the free drug as indicated by our previous studies [35, 41]. In our first experiment the cells were treated for 6 h with the conjugates, then the cells were washed and incubated for another 66 h (cytostatic effect). In the second experiment the conjugates were added to the cells for 72 h (cytotoxic effect). The IC_50_ values are summarized in Fig 8.

As expected, free daunomycin was equally toxic to the cells. Conjugate **1,** containing the least stable ring did not show any cytostatic effect in the concentration range measured. After 72 h, a slight increase of the cytotoxic effect was observed, which was more pronounced on HT-29 cells. This unique behaviour suggests that conjugate **1** might internalize *via* integrin receptors after the formation of the *iso*Asp derivative. Conjugate **1** has a cyclic NGR part with a 15-membered ring in contrast with the other compounds having 17- or 18-membered rings in their structure. Therefore, it might be concluded that this small ring size does not allow appropriate receptor binding to CD13.

In contrast, conjugate **2** (containing a disulfide bridge) showed preferential toxicity to HT-1080 cells in the cytostatic experiments, resulting in the highest selectivity. The antitumor effect of **2** increased in time (72 h treatment), but its selectivity decreased significantly. In comparison to conjugates **3** (thioether linkage) and **5** (amide bond), both containing free Lys in the cycle, conjugate **5** had slightly better activity against HT-1080 cells. However, the selectivity of conjugate **5** to CD13 receptors (as judged by the relative toxicity against the two cell lines) was not significant after 6 h treatment, whereas it was significantly more toxic to HT-1080 cells in the 72 h experiment. This can also be explained by its higher chemostability that results in longer exposure the cells to the unmodified NGR peptide–drug conjugate. In contrast, their analogs in which the drug molecule is connected to the side chain of Lys in the cycle (conjugates **4** and **6**) were more specific but less toxic, especially in the cytotoxicity experiments. Interestingly, there was no correlation between the specificity and the chemostability of the conjugates. To better understand the observed differences in the cytostatic/cytotoxic effects, lysosomal degradation and cellular uptake of the conjugates were studied.

### Lysosomal degradation

**Fig 9:**
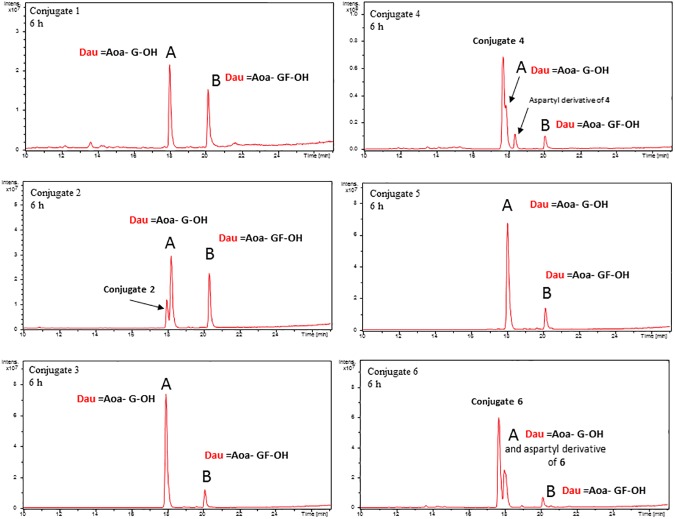
Lysosomal degradation of daunomycin-conjugates after 6 h.

Conjugates for drug targeting with receptor-binding peptides may enter the cells by receptor mediated endocytosis. Endocytic vesicles fuse with lysosomes, which is followed by the degradation of the conjugates by lysosomal enzymes. Cathepsin B is one of the main lysosomal enzymes overexpressed in cancer cells. Therefore, Cathepsin B labile spacers (*e*.*g*. GFLG tetrapeptide) are often incorporated between the drug and targeting moiety [41]. We used a rat lysosomal homogenate to study the degradation of conjugates and to follow the release of drug containing metabolites (Fig 9, S9–S14 Figs). The results indicated that conjugates **1**, **3** and **5** decomposed within 6 h. The main Dau containing metabolite was Dau = Aoa-Gly-OH that binds efficiently to DNA [35]. In addition, a minor peak corresponding to Dau = Aoa-Gly-Phe-OH could also be detected. The peak corresponding to Dau = Aoa-GF-OH was the highest in case of conjugate **1**. In contrast to these conjugates, decomposition of the disulfide bond containing conjugate (**2)** was much slower. After 6 h the presence of the starting compound could be detected and the peak of Dau = Aoa-Gly-Phe-OH was also high. This slow degradation might be one of the reasons why conjugate **2** is less toxic than conjugate **5,** which has the same ring size. Interestingly, branching conjugates **4** and **6** decomposed very slowly probably because of steric hindrance. After 6 h incubation the main components were still the intact conjugates **4** and **6,** which might have weak binding activity to DNA, explaining the low toxicity of these conjugates. After 72 h only Dau = Aoa-Gly-OH could be detected in cases of all conjugates.

### Cellular uptake

**Fig 10:**
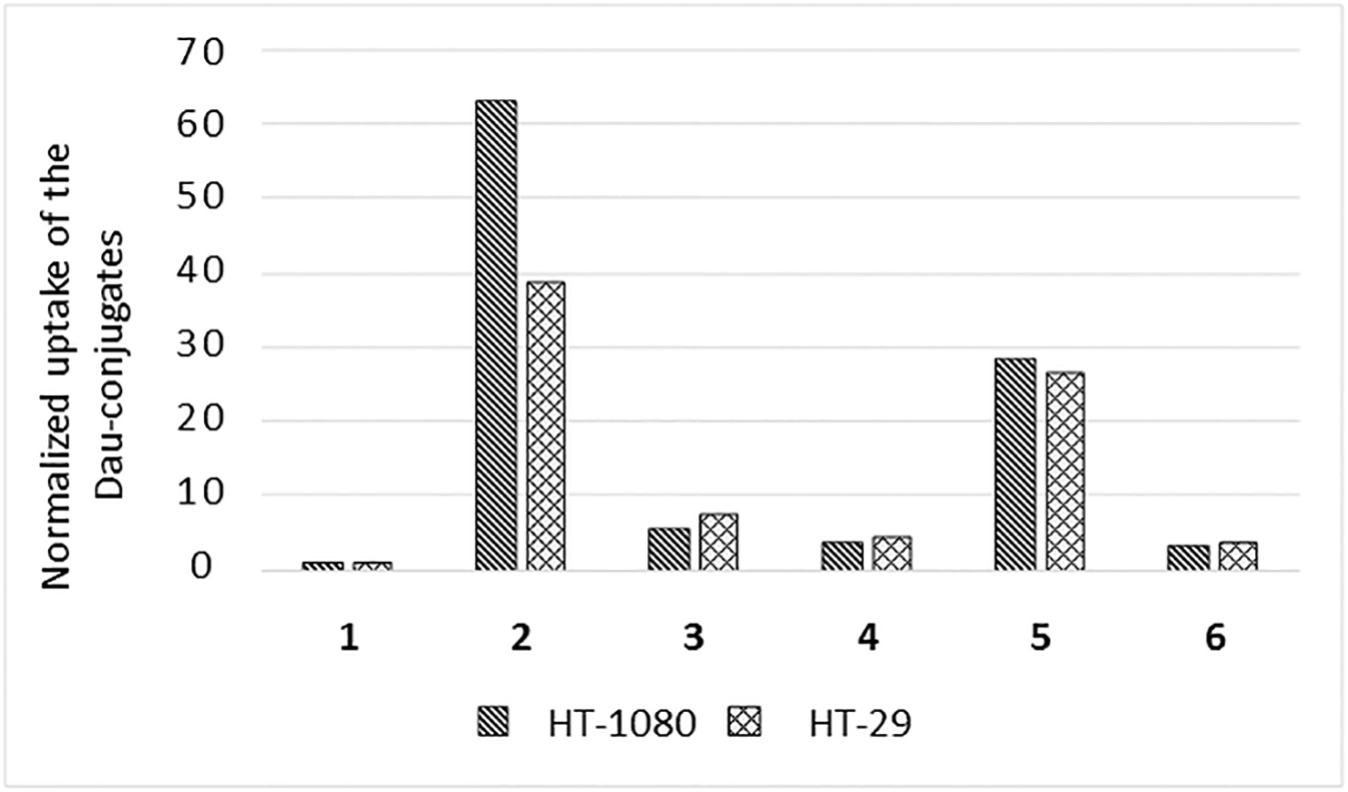
Direct uptake of daunomycin-conjugates. Normalized uptake values indicate the fold increase of the measured fluorescent units compared to the autofluorescence of empty cells.

As daunomycin-conjugates are fluorescent, we were able to follow their cellular uptake with a flow cytometer (Fig 10, S15 Fig). Conjugates **2** and **5** (both having 17-atom ring size in NGR peptide) showed higher accumulation after 6 h incubation than the other conjugates, in accordance with their higher activity in the cytostatic experiments. Significantly, conjugate **2,** which was selective in the short term cytostatic experiments, showed higer accumulatoin in HT-1080 cells. The higher uptake of **2** compared to **5** might partly compensate its slower degradation resulting in almost similar bioactivity. Conjugate **3** showed a moderate uptake that was a bit more pronounced in HT-29 cells. We speculate that the high cytostatic effect of **3** is explained by its fast decomposition resulting in the active Dau = Aoa-Gly-OH metabolite. Conjugate **1** did not show significant cellular uptake. This observation suggests that conjugate **1** has no affinity to CD13 receptors. However, after deamidation the formed *iso*DGR might be recognized by RGD integrin receptor, that results in moderate cytotoxicity in both cell lines during a longer treatment. Conjugates **4** and **6** showed low cellular uptake by both types of cell during the 6 h treatment. Taking also the slow metabolite release into account, this observation explains the lack of significant cytostatic effect.

## Conclusions

Six new cyclic NGR peptide–daunomycin conjugates were prepared with different structure in terms of conjugation sites and applied linkage in the cycle. Chemostability (deamidation of Asn) of the conjugates and the free cyclic peptides [32] was found to be different. However, in most cases no correlation between the chemostability and selectivity or dual acting effect of conjugates could be identified. Therefore, further experiments are needed to analyse the binding affinity of conjugates to different receptors. Nevertheless, we can conclude that the conjugates in which the drug molecule is attached through *C*-terminal elongation of cyclic NGR peptides (conjugates **2**, **3**, **5**) have higher antitumor activity against both cell lines, irrespectively of the bond in the cycle, as compared to branched conjugates **4** and **6.** Conjugates **4** and **6** showed higher selectivity to HT-1080 cells. Therefore, conjugates **2**, **3** and **5** are suitable for dual targeting while conjugates **4** and **6** can be used for selective CD13 targeting. We also find that the antitumor activity of the NGR peptide–drug conjugates depends on complex mechanisms including cellular uptake, enzyme degradation and NGR-*iso*DGR rearrangement. In addition, conjugates **2**, **3** and **5** showed similar antitumor activity against HT-29 cells *in vitro* as the previously prepared GnRH-III–Dau conjugate [42] that had significant and better *in vivo* tumor growth inhibition effect compared to the free drug [36]. These results warrant the study of the *in vivo* antitumor effect of the most active NGR–Dau conjugates alone, or in combination with the GnRH–drug conjugate.

## Supporting information

S1 Fig Charactheristics of isopropylidene protected aminooxyacetic acid. 1H and 13C NMR spectra were recorded on a Bruker-Avance 200 MHz spectrometer in DMSO-d6 at room temperature (303 K). Chemical shifts (δ) are given in parts per million (ppm) units relatively to the internal standard TMS (δ = 0.00 for 1 H, δ = 0.00 for 13C). ^1^H NMR (200 MHz, DMSO-d6): δ 4.42 (s, 2H, CH_2_), 1.80 (s, 3H, CH_3_), 1.77 (s, 3H CH_3_); ^13^C NMR (62,9 MHz, DMSO-d6) δ 172.34, 156.39, 70.58, 22.14, 16.46 ppm.
(PDF)

S2 Fig Chemostability and Asp control of Dau = GFLGK([CH_2_CO-NGRC]GG)-NH_2_ (1). (PDF)

S3 Fig Chemostability and Asp control of Dau = GFLGK(Ac-[CNGRC]GG)-NH_2_ (2). (PDF)

S4 Fig Chemostability and Asp control of Dau = GFLGK([CH_2_CO-KNGRC]GG)-NH_2_ (3). (PDF)

S5 Fig Chemostability and Asp control of [CH_2_CO-K(Dau = Aoa-GFLG)NGRC]-NH_2_ (4). (PDF)

S6 Fig Chemostability and Asp control of Dau = GFLGK([KNGRE]GG)-NH_2_ (5). (PDF)

S7 Fig Chemostability and Asp control of [K(Dau = Aoa-GFLG)NGRE]-NH_2_ (6). (PDF)

S8 Fig Characterization of CD13 expression by flow cytometry. Expression of CD13 in HT1080 (A) and HT-29 cells (B). Expression was followed using the FITC-conjugated CD13-specific OKM13 antibody (black); red histograms represent the isotype control.
(PDF)

S9 Fig Lysosomal degradation of Dau = GFLGK([CH_2_CO-NGRC]GG)-NH_2_ (1). (PDF)

S10 Fig Lysosomal degradation of Dau = GFLGK(Ac-[CNGRC]GG)-NH_2_ (2). (PDF)

S11 Fig Lysosomal degradation of D au = GFLGK([CH_2_CO-KNGRC]GG)-NH_2_ (3). (PDF)

S12 Fig Lysosomal degradation of [CH_2_CO-K(Dau = Aoa-GFLG)NGRC]-NH_2_ (4). (PDF)

S13 Fig Lysosomal degradation of Dau = GFLGK([KNGRE]GG)-NH_2_ (5). (PDF)

S14 Fig Lysosomal degradation of [K(Dau = Aoa-GFLG)NGRE]-NH_2_ (6). (PDF)

S15 Fig Cell uptake measurements with FACS. Accumulation of daunomycin-conjugates in (A) HT1080 and in (B) HT29 cells. Uptake of compound **1** (yellow); **2** (red); **3** (light green); **4** (dark green); **5** (purple); **6** (light blue). Empty control (the autofluorescence of the cells) is indicated with dark blue. Side scatter versus forward scatter values for (C) HT1080 and for (D) HT29 cells.
(PDF)
