# Supplementary figures and images for: NGR-peptide−drug conjugates with dual targeting properties
Source: PLoS One. 2017 Jun 2;12(6):e0178632. doi: 10.1371/journal.pone.0178632 (PMC5456102; doi:10.1371/journal.pone.0178632)

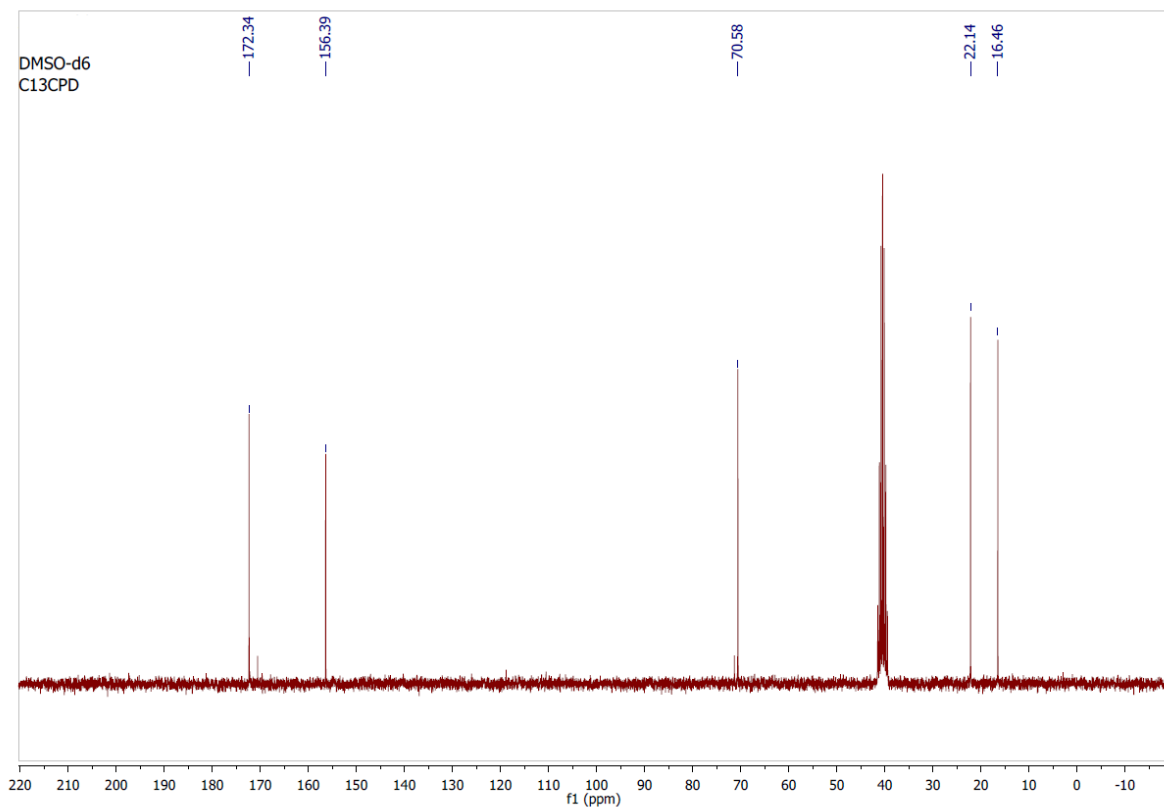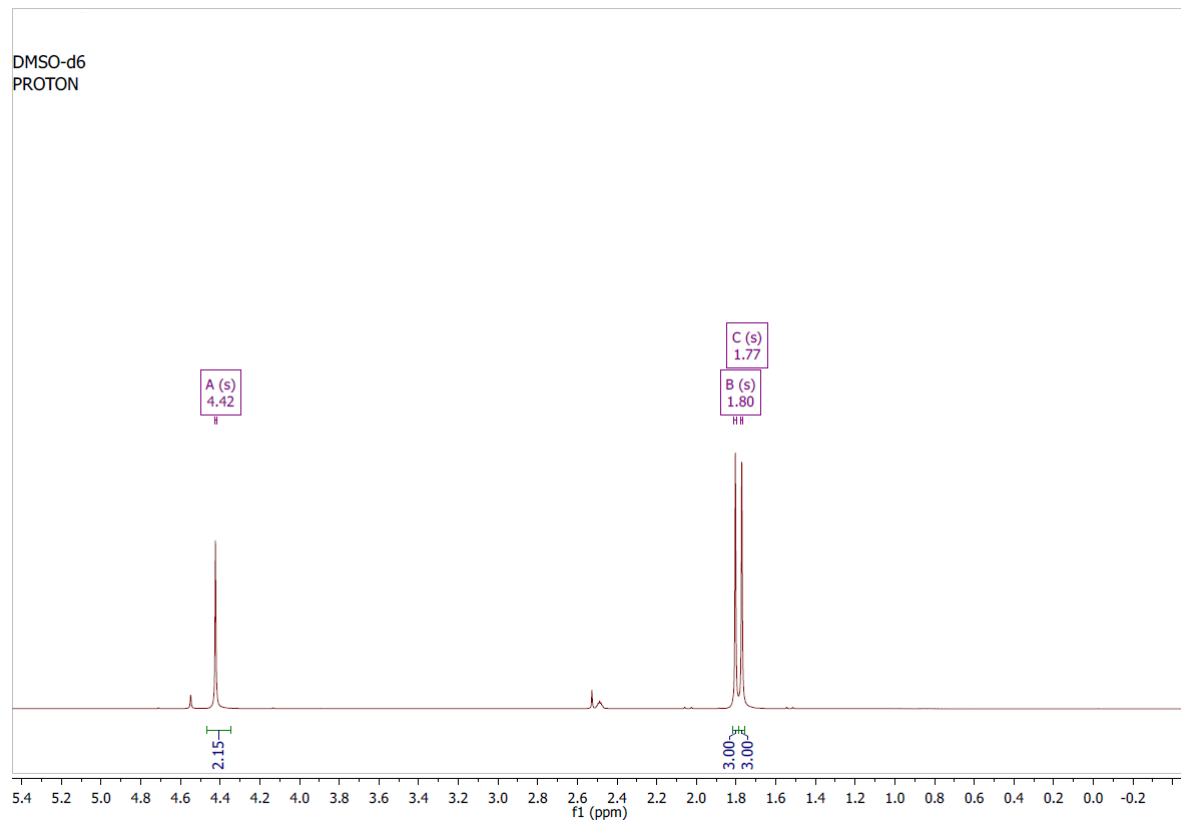

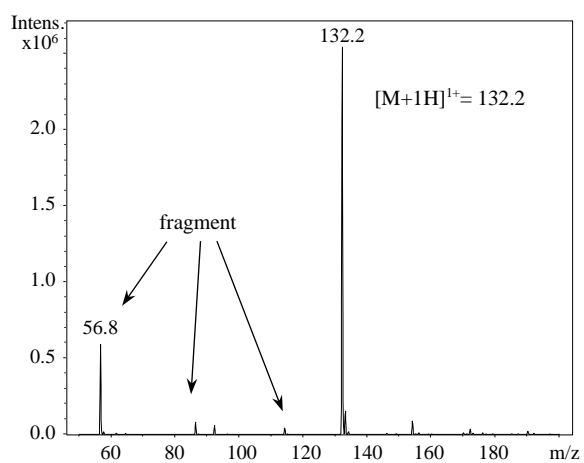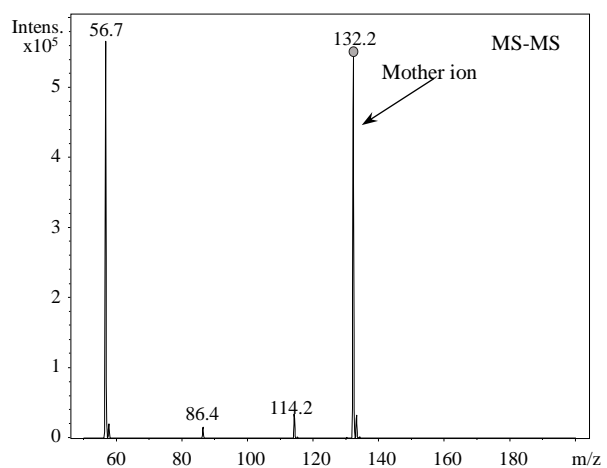

Supplement: S1 Fig — 1H and 13C NMR spectra were recorded on a Bruker-Avance 200 MHz spectrometer in DMSO-d6 at room temperature (303 K). Chemical shifts (δ) are given in parts per million (ppm) units relatively to the internal standard TMS (δ = 0.00 for 1 H, δ = 0.00 for 13C). 1H NMR (200 MHz, DMSO-d6): δ 4.42 (s, 2H, CH2), 1.80 (s, 3H, CH3), 1.77 (s, 3H CH3); 13C NMR (62,9 MHz, DMSO-d6) δ 172.34, 156.39, 70.58, 22.14, 16.46 ppm. (PDF) [file pone.0178632.s001.pdf]

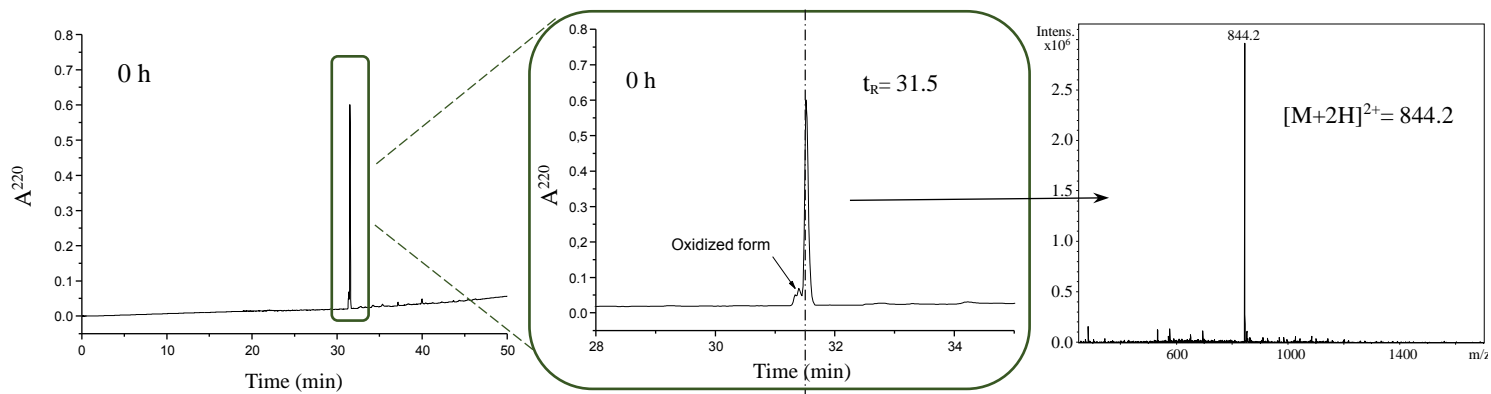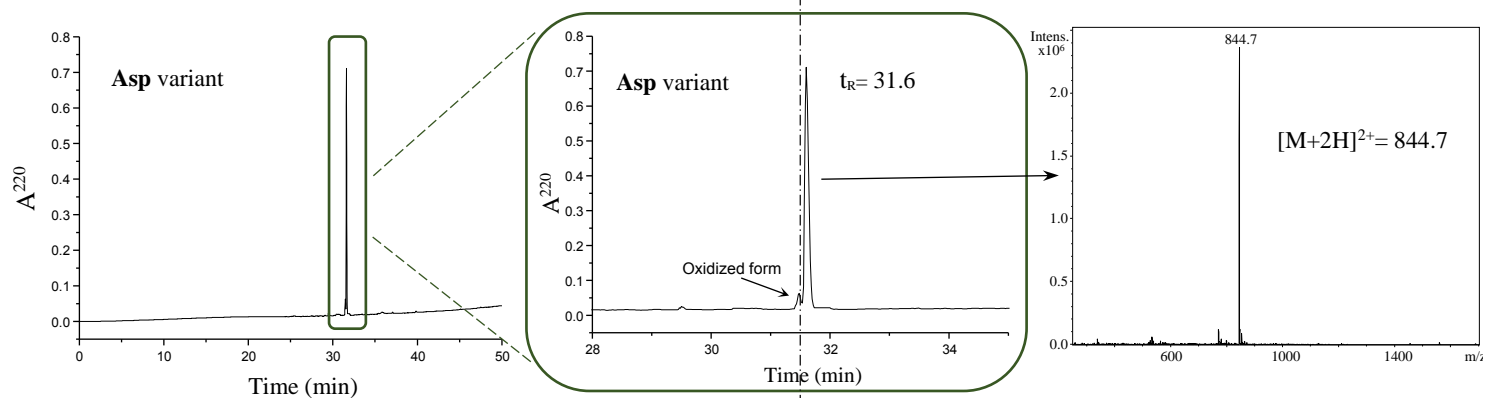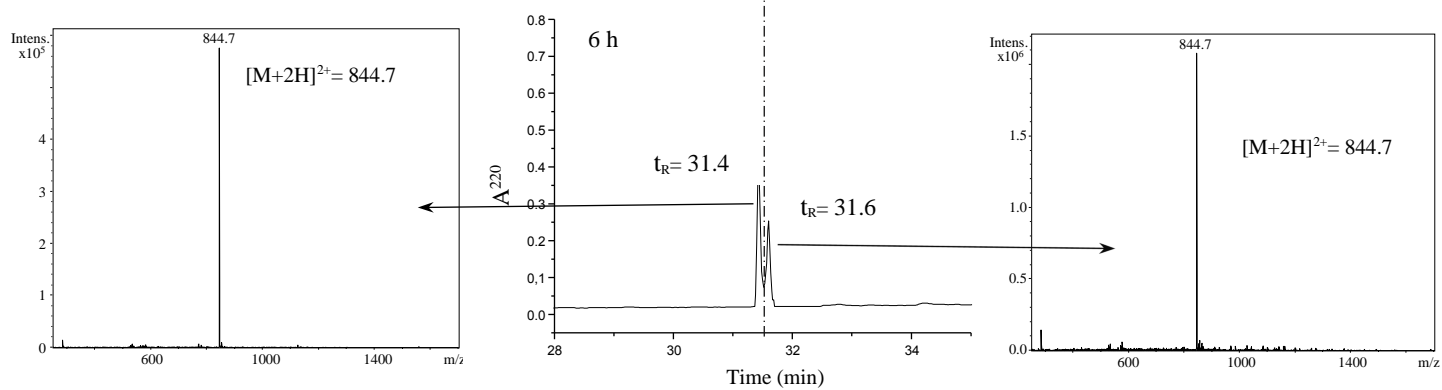

Supplement: S2 Fig — (PDF) [file pone.0178632.s002.pdf]

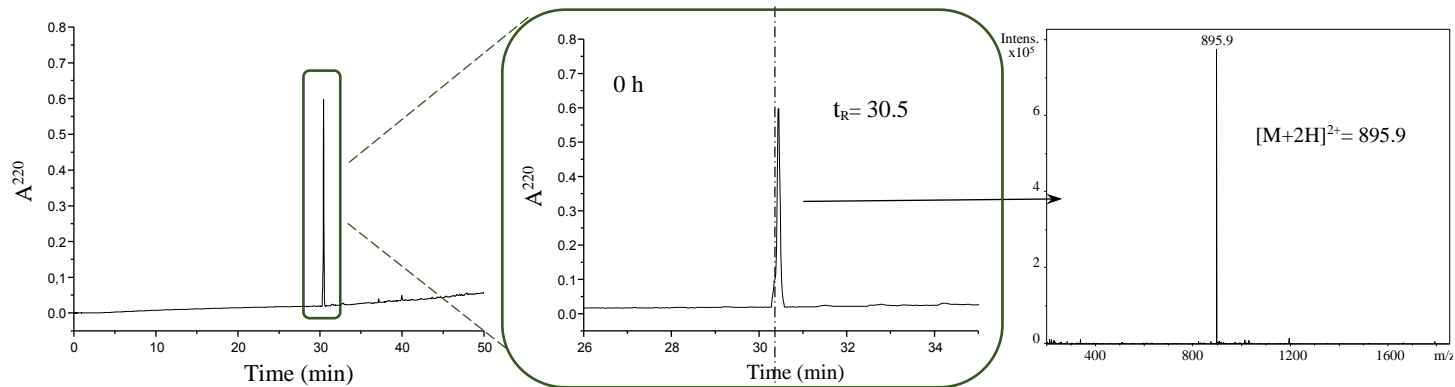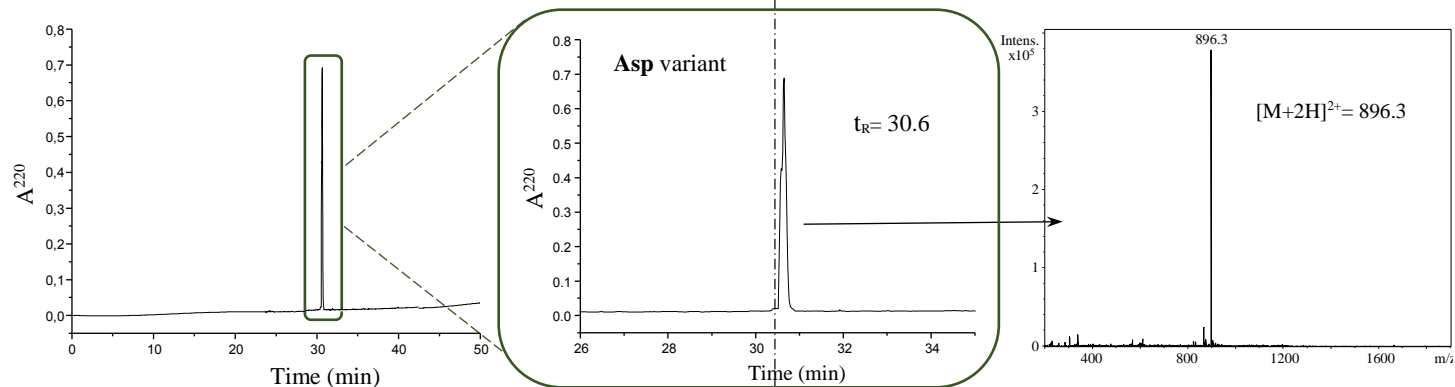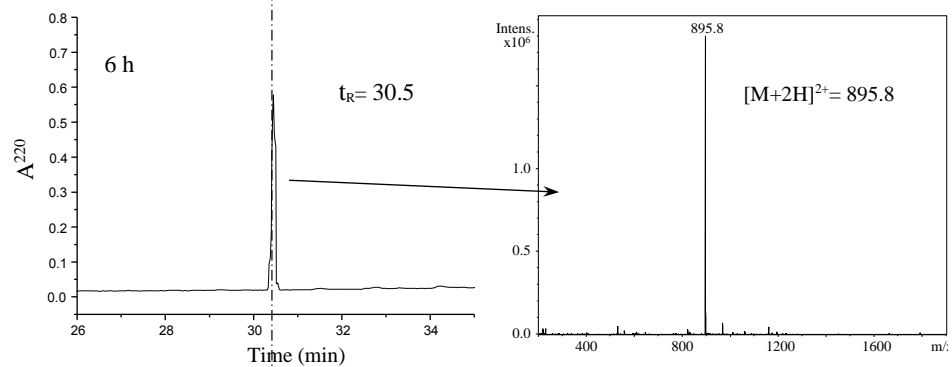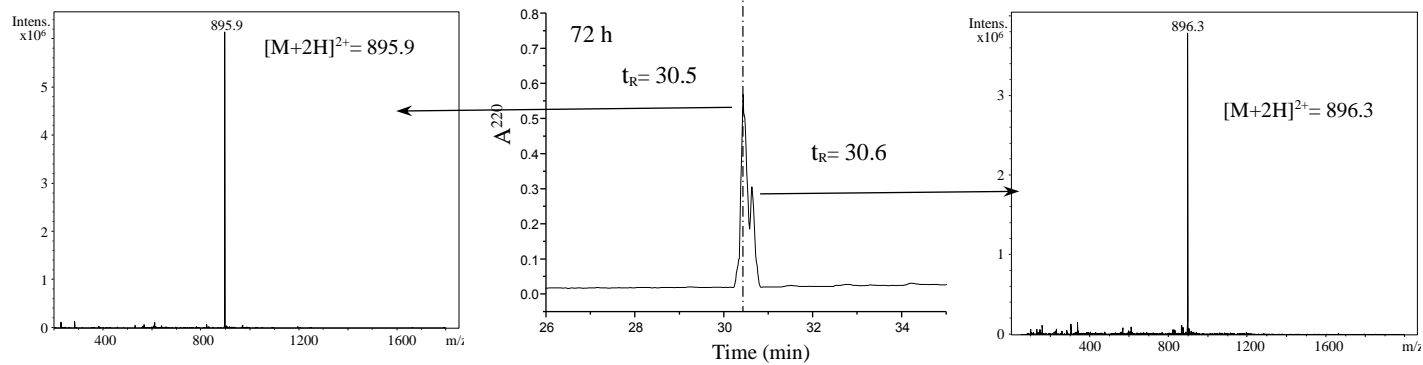

Supplement: S3 Fig — (PDF) [file pone.0178632.s003.pdf]

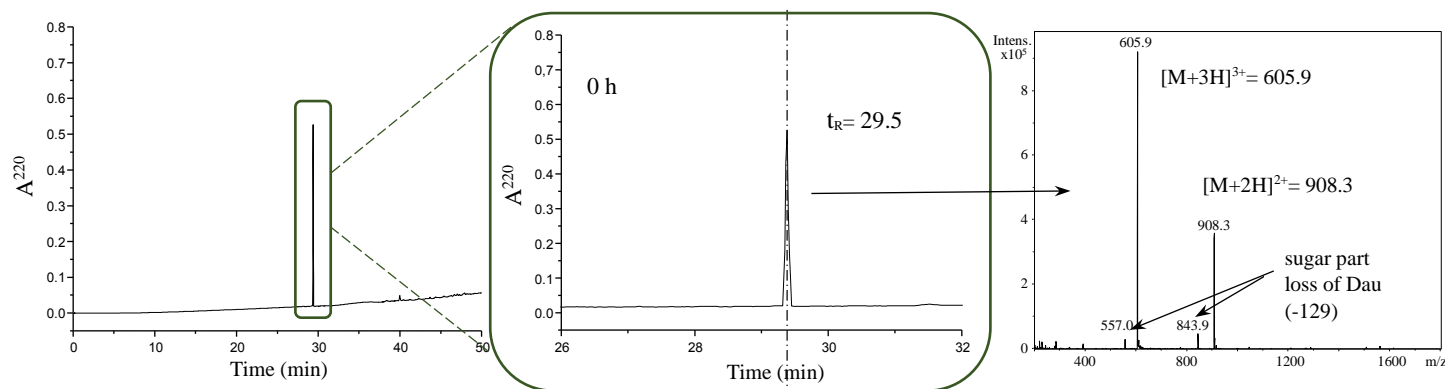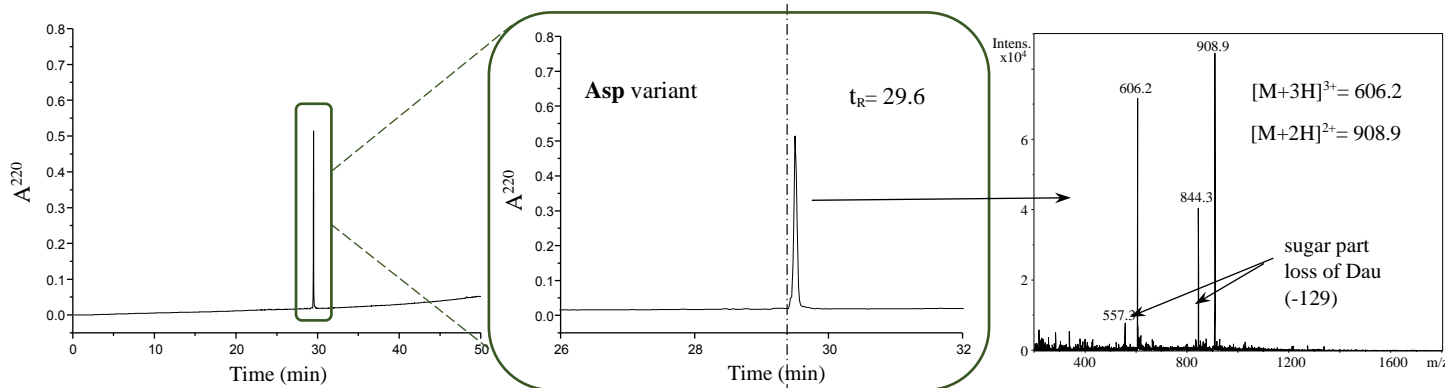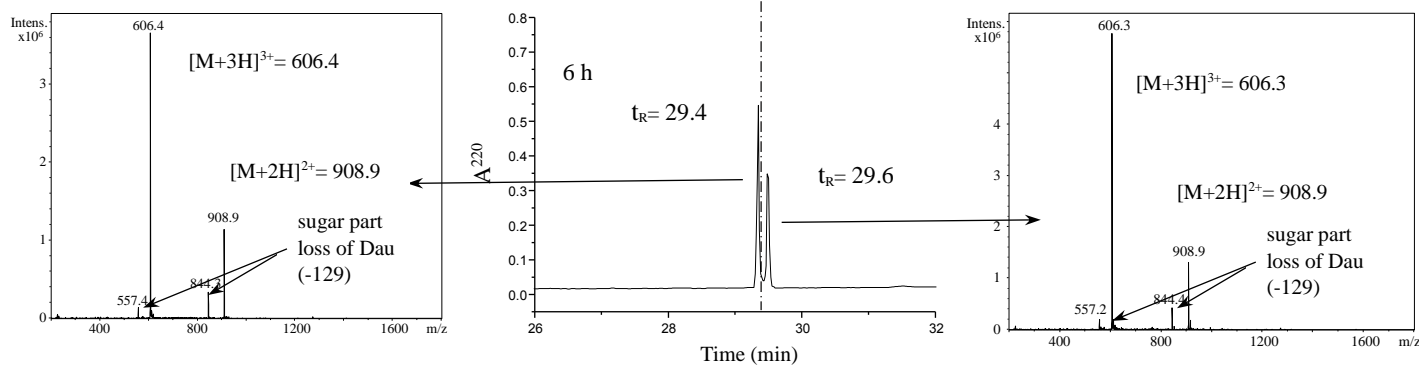

Supplement: S4 Fig — (PDF) [file pone.0178632.s004.pdf]

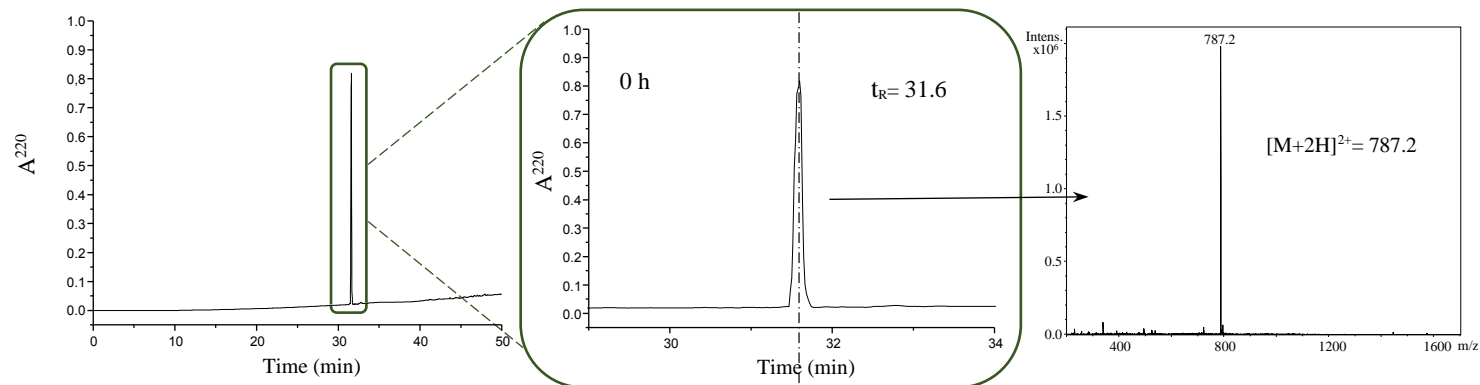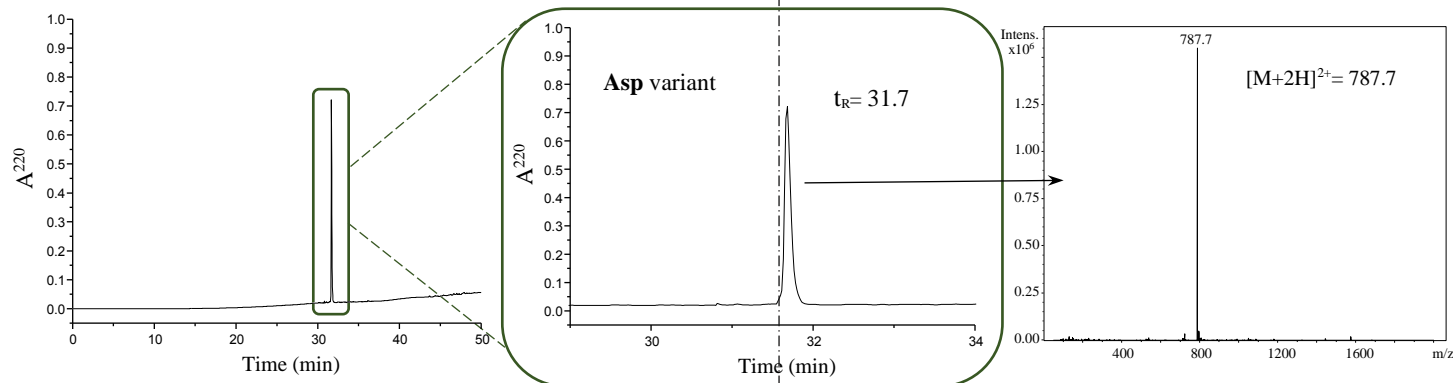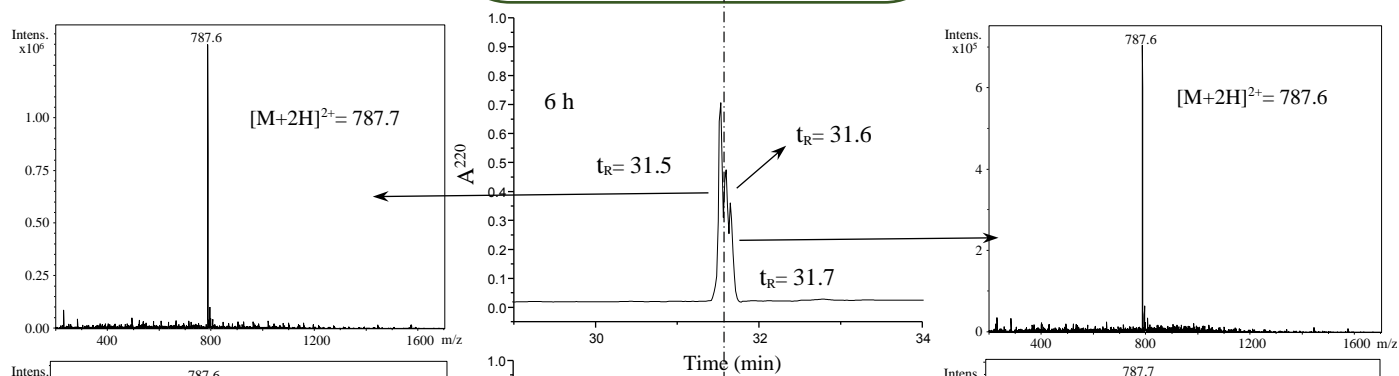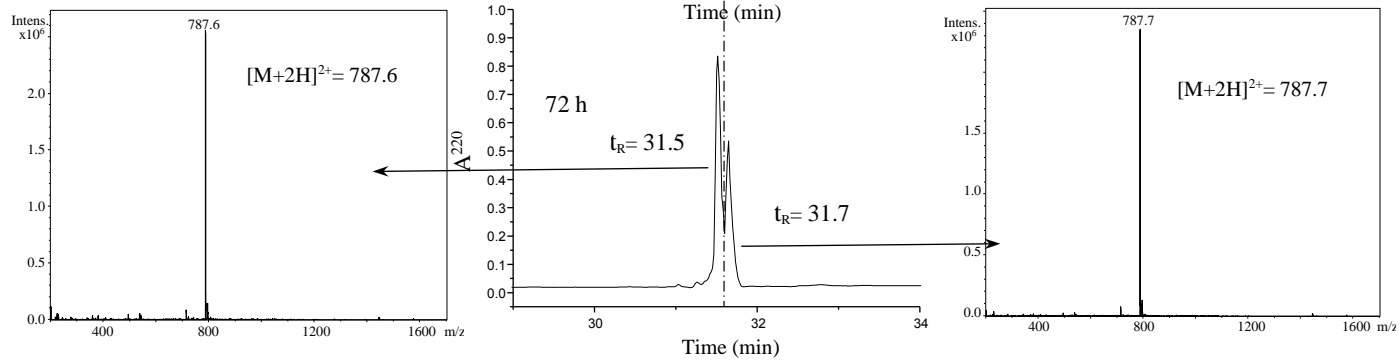

Supplement: S5 Fig — (PDF) [file pone.0178632.s005.pdf]

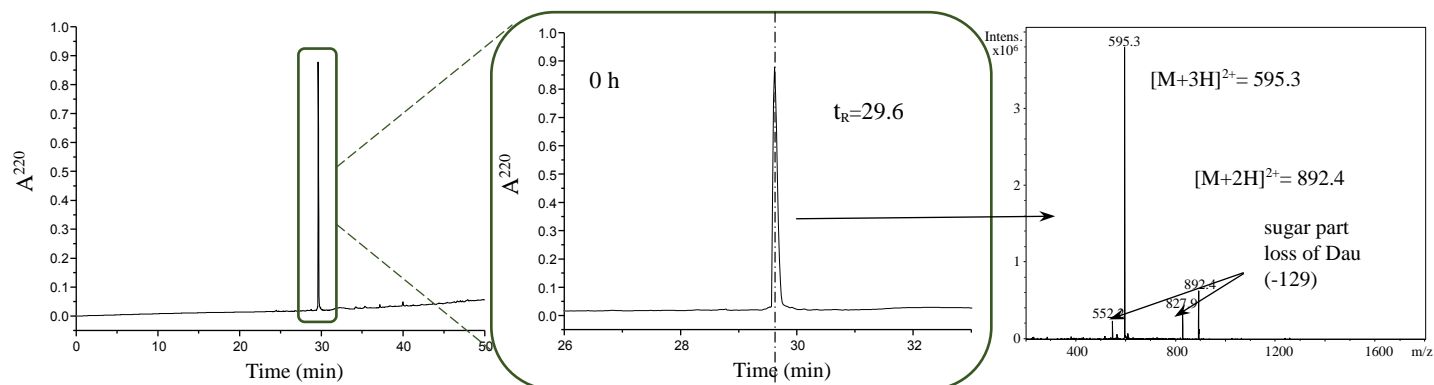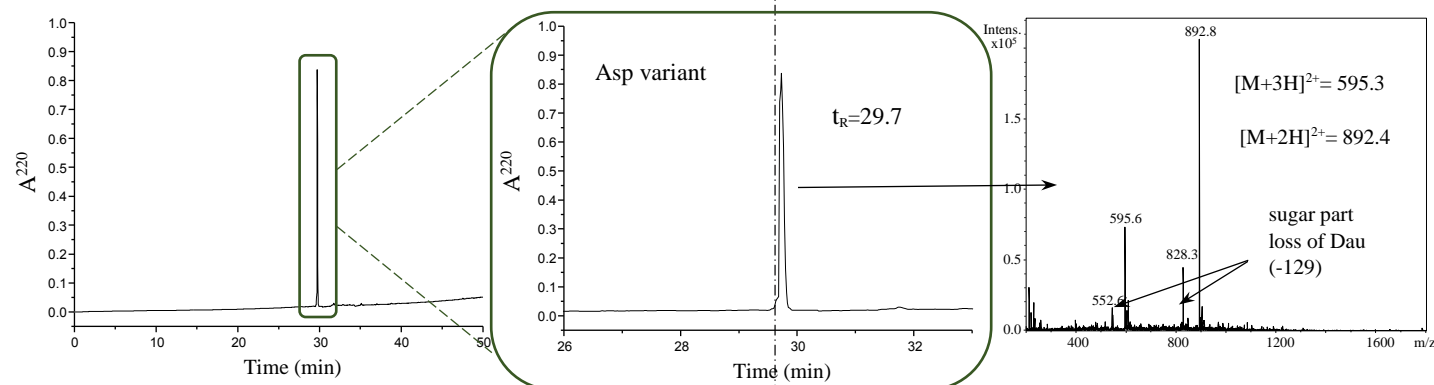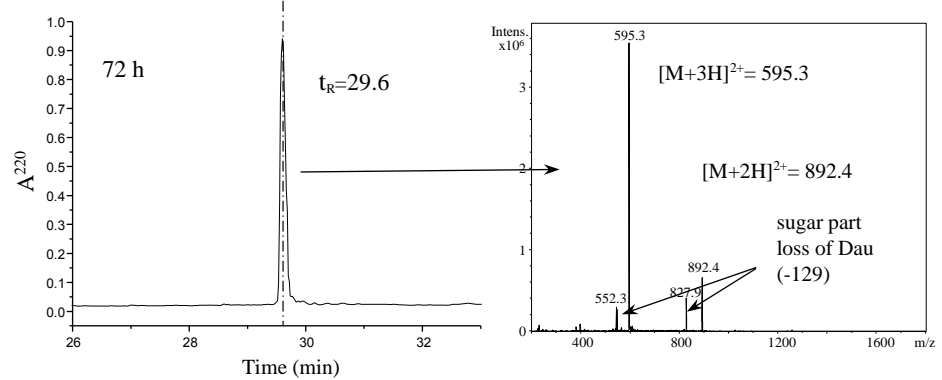

Supplement: S6 Fig — (PDF) [file pone.0178632.s006.pdf]

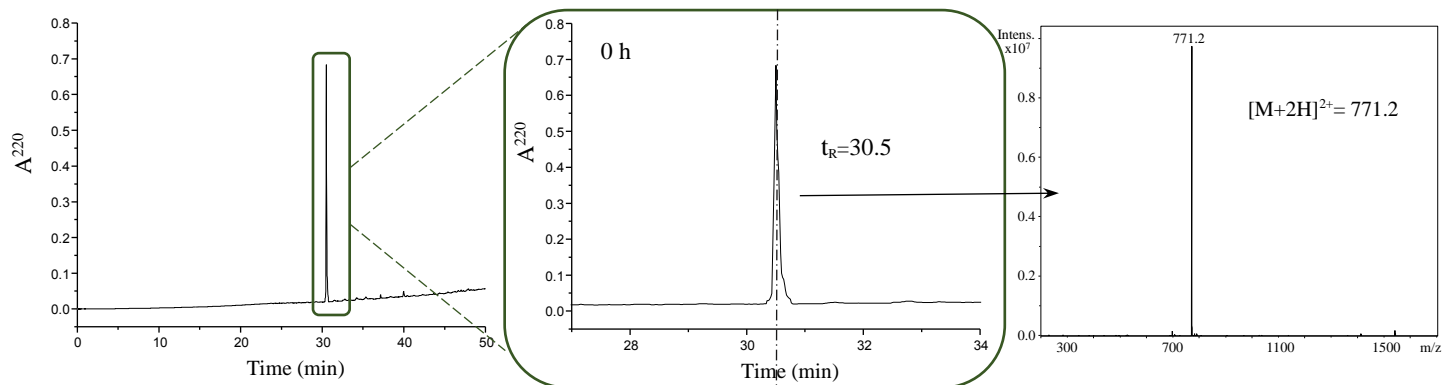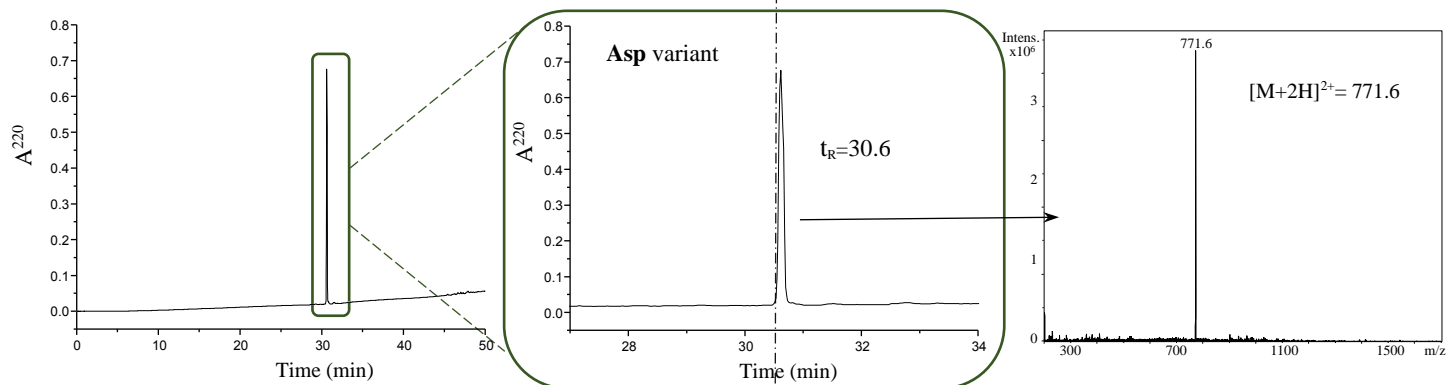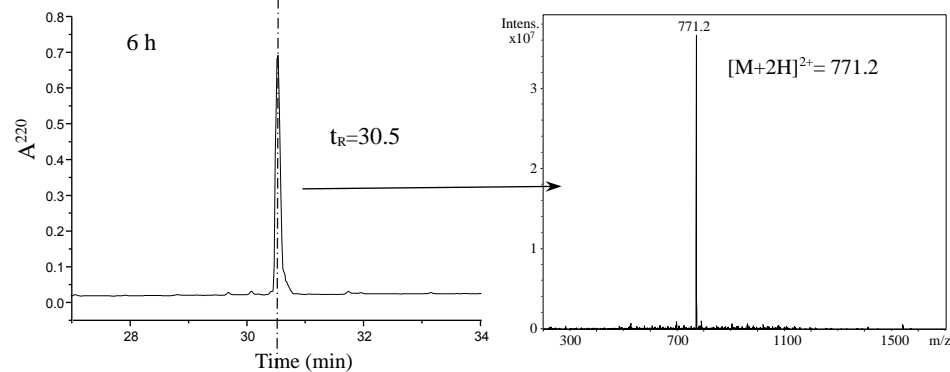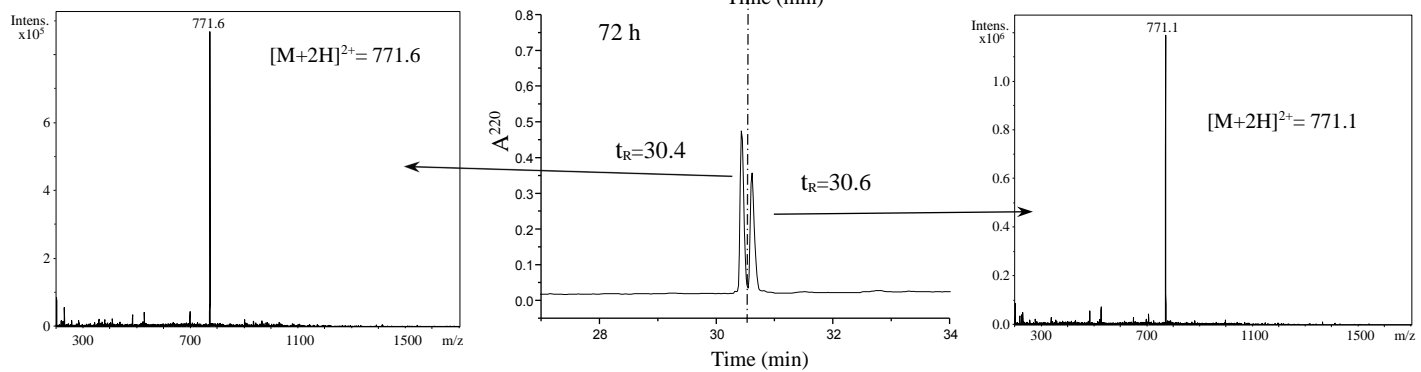

Supplement: S7 Fig — (PDF) [file pone.0178632.s007.pdf]

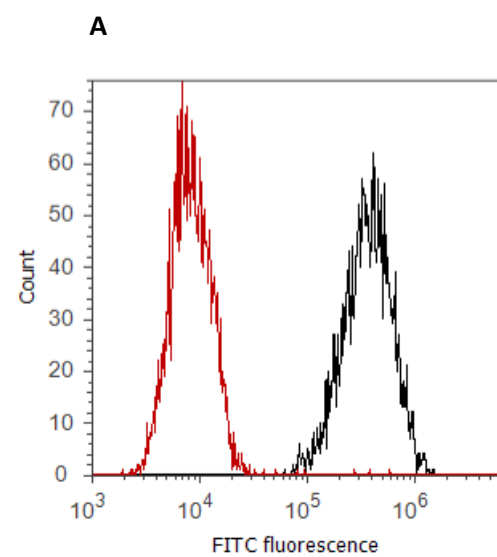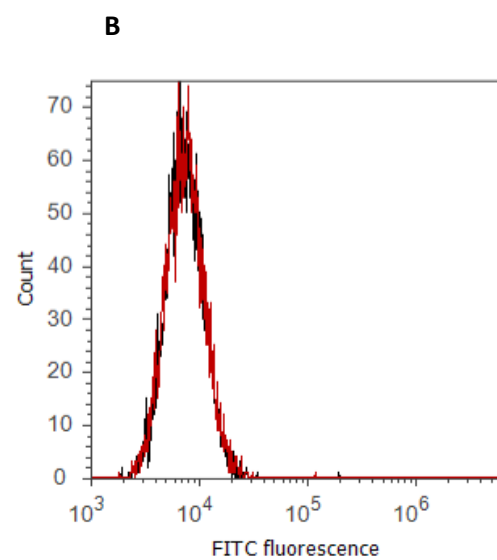

Supplement: S8 Fig — Expression of CD13 in HT1080 (A) and HT-29 cells (B). Expression was followed using the FITC-conjugated CD13-specific OKM13 antibody (black); red histograms represent the isotype control. (PDF) [file pone.0178632.s008.pdf]

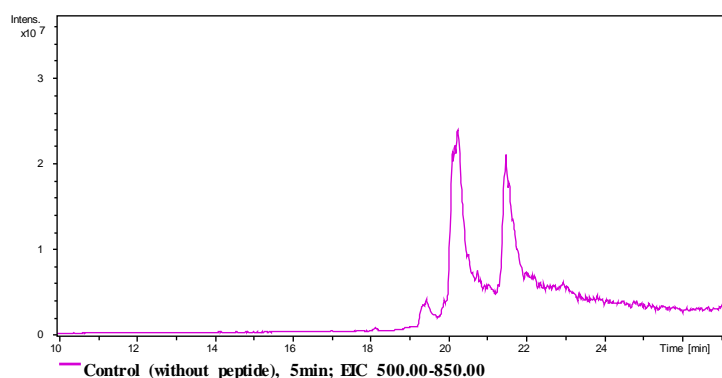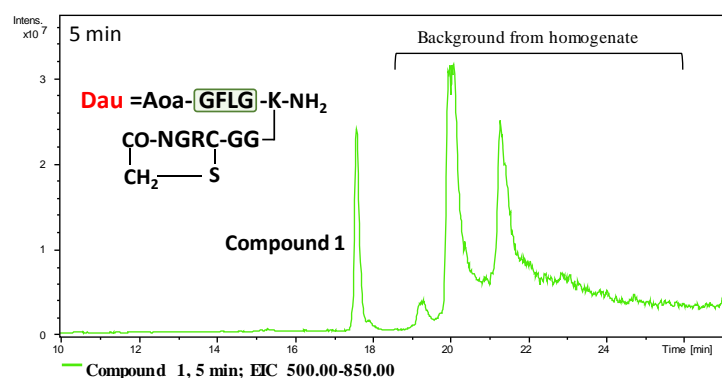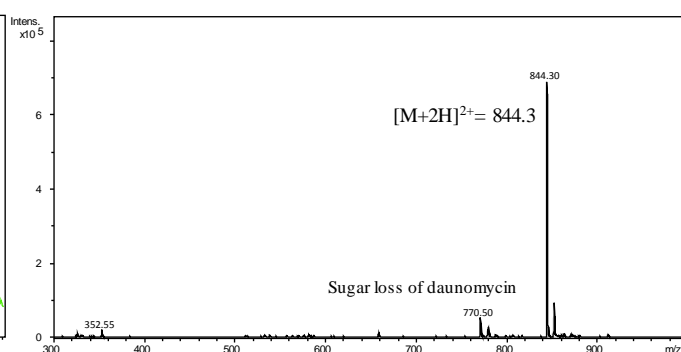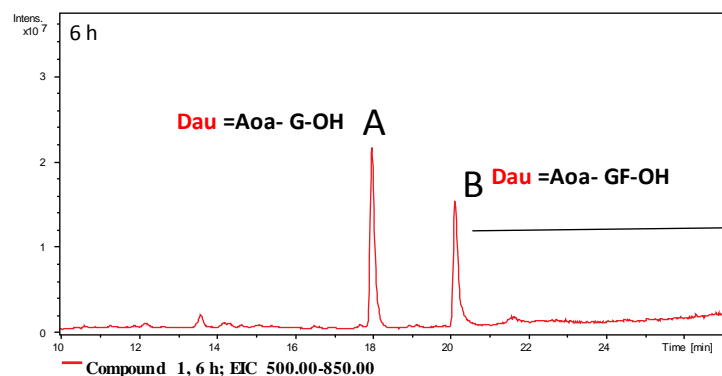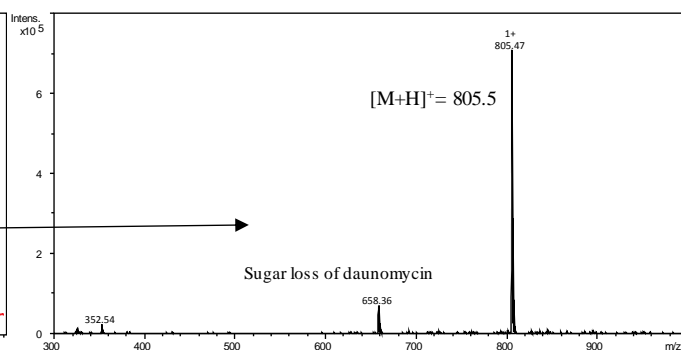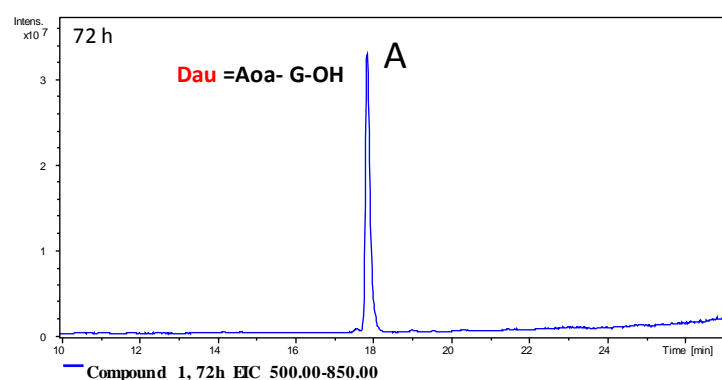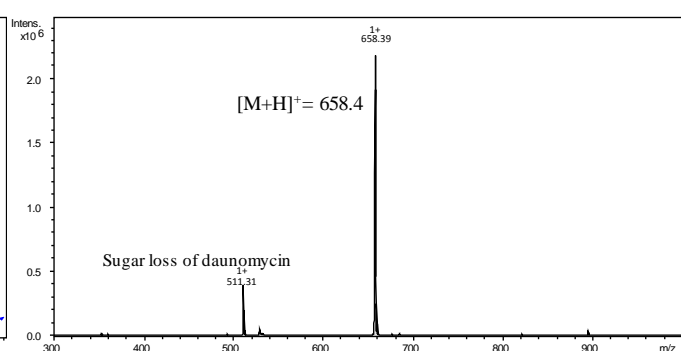

Supplement: S9 Fig — (PDF) [file pone.0178632.s009.pdf]

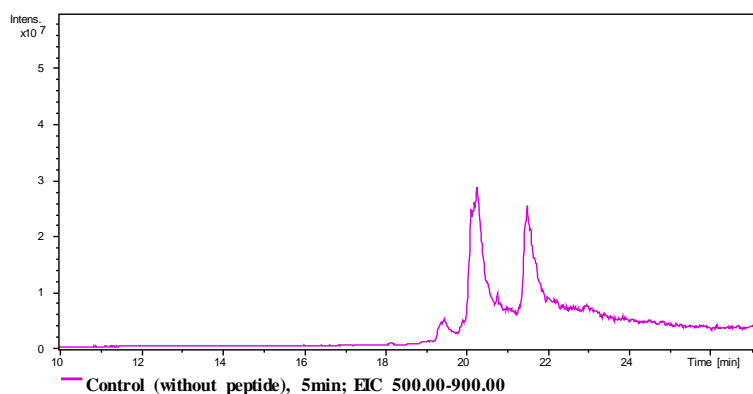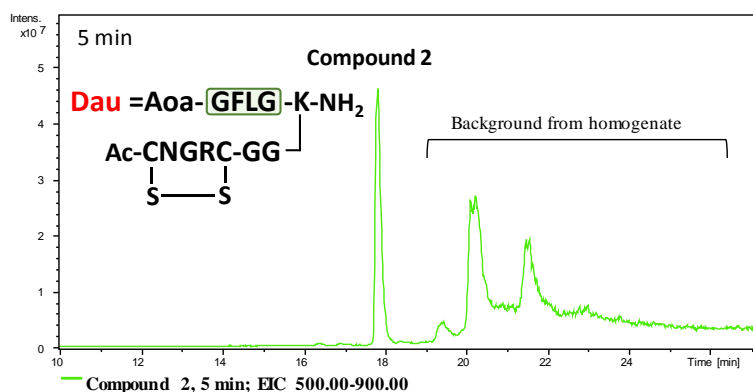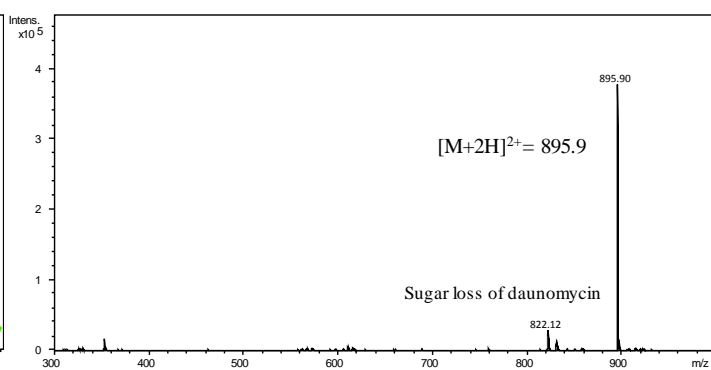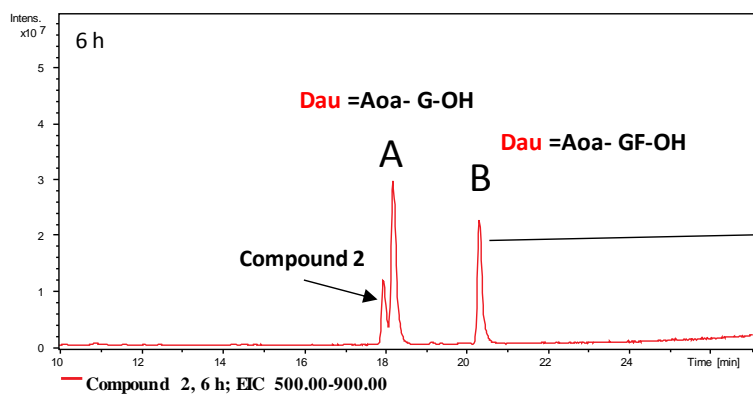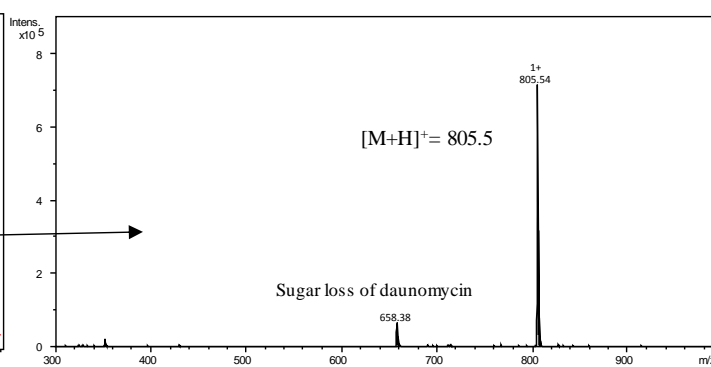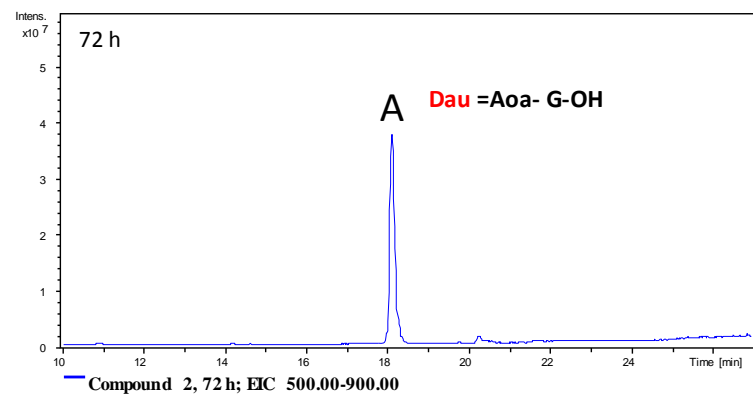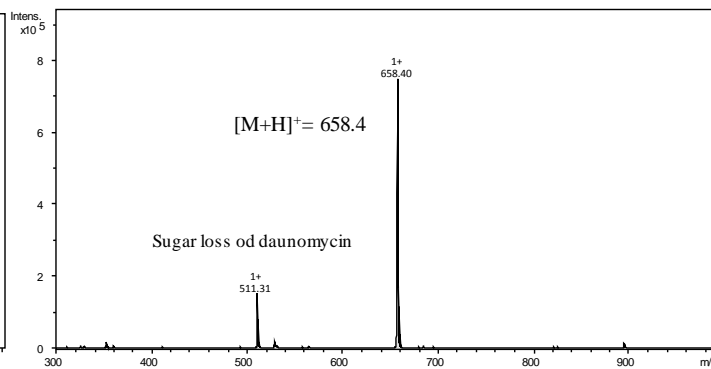

Supplement: S10 Fig — (PDF) [file pone.0178632.s010.pdf]

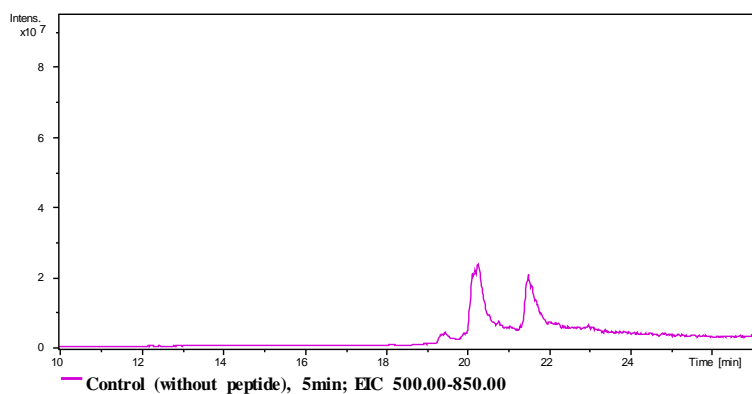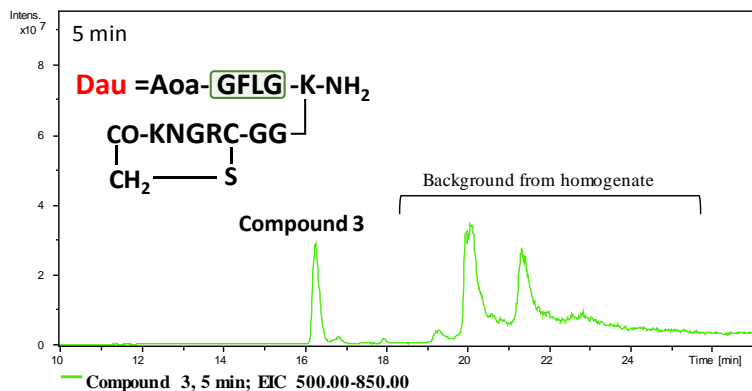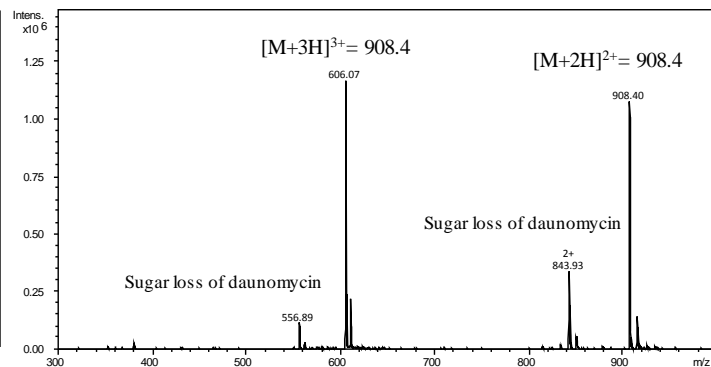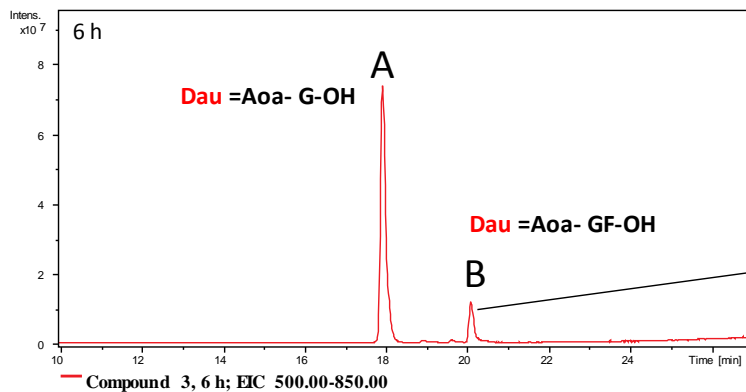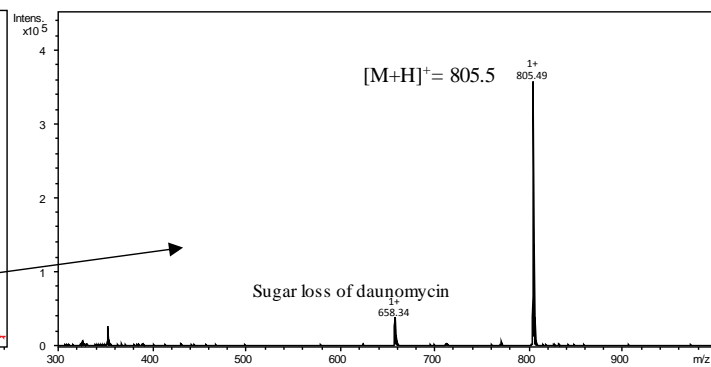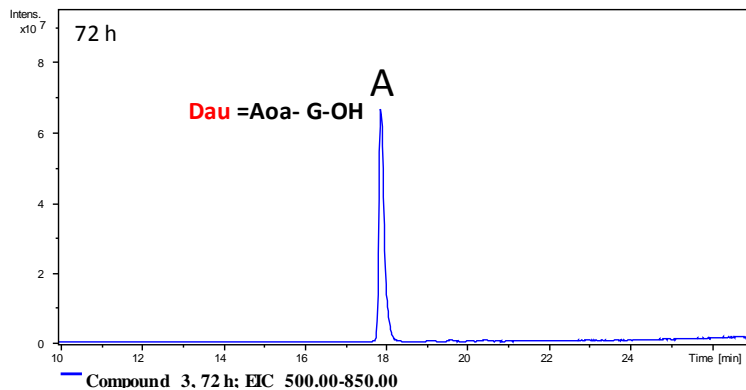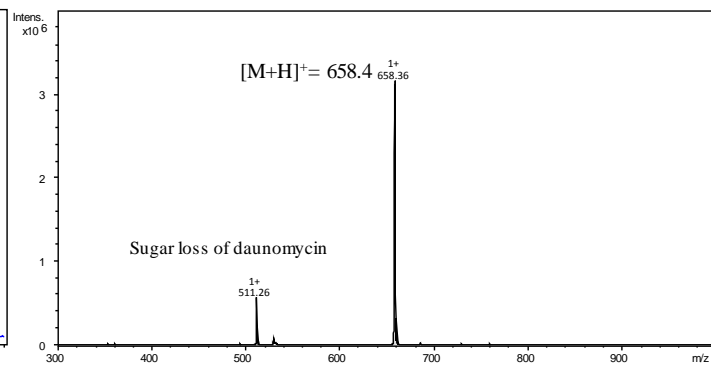

Supplement: S11 Fig — (PDF) [file pone.0178632.s011.pdf]

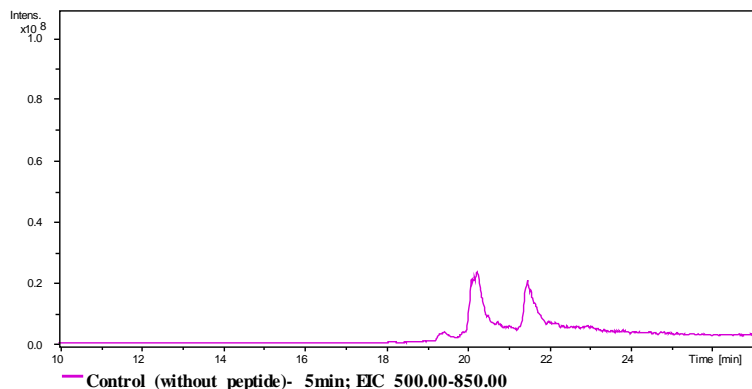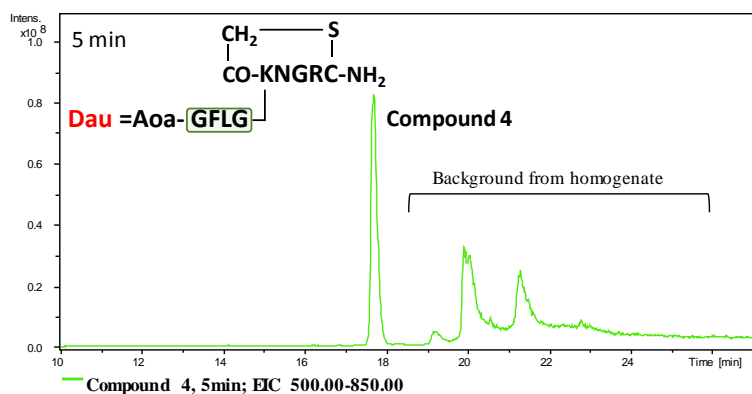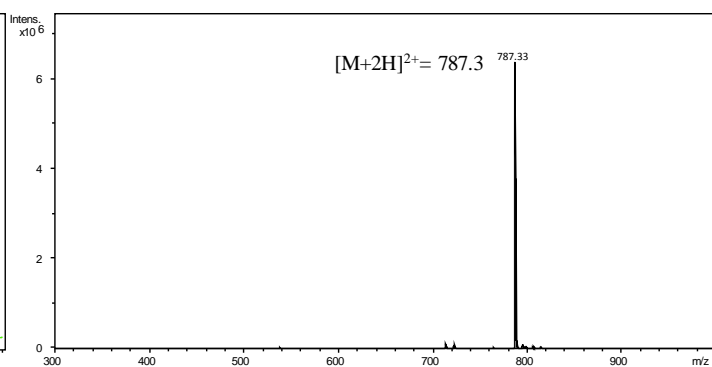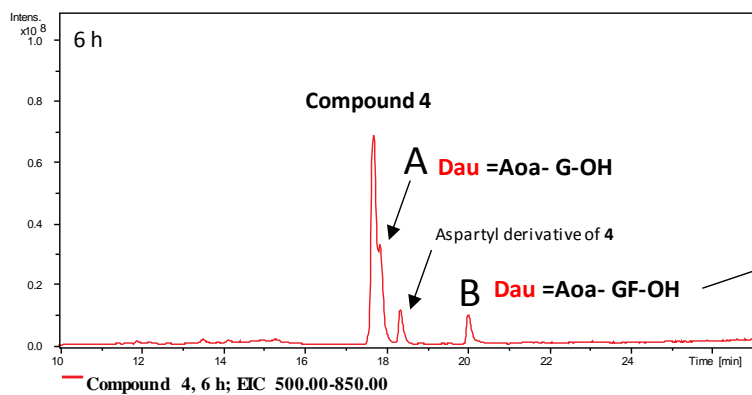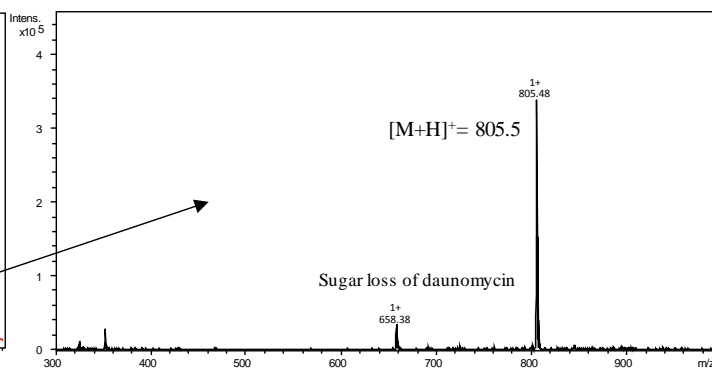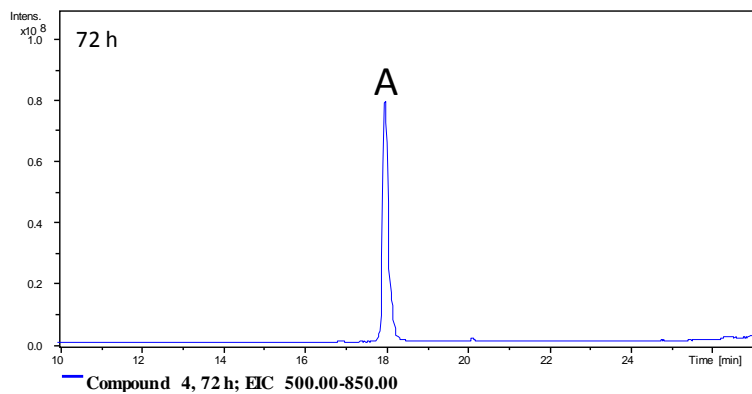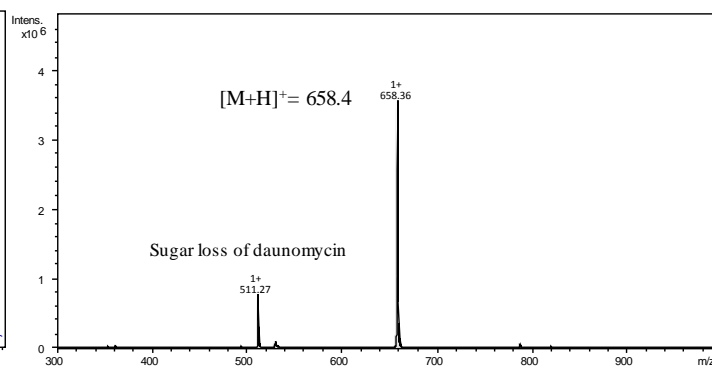

Supplement: S12 Fig — (PDF) [file pone.0178632.s012.pdf]

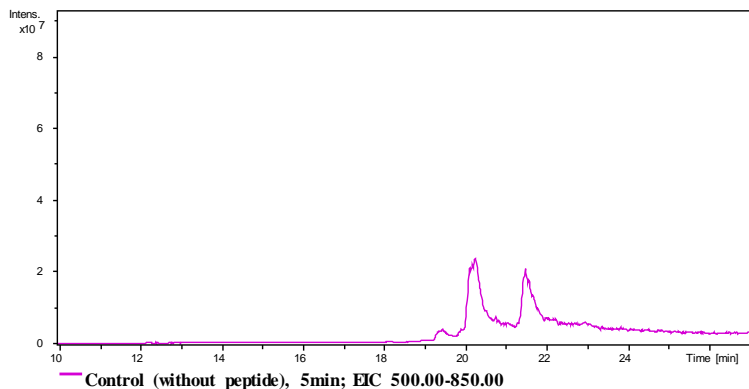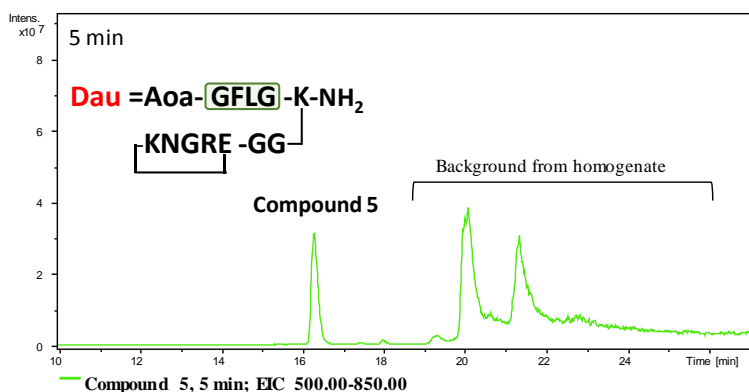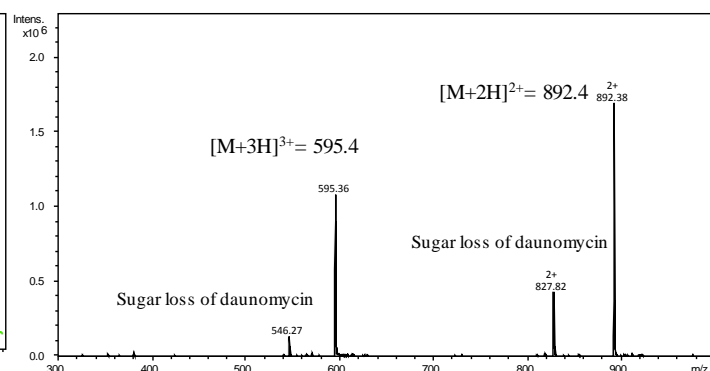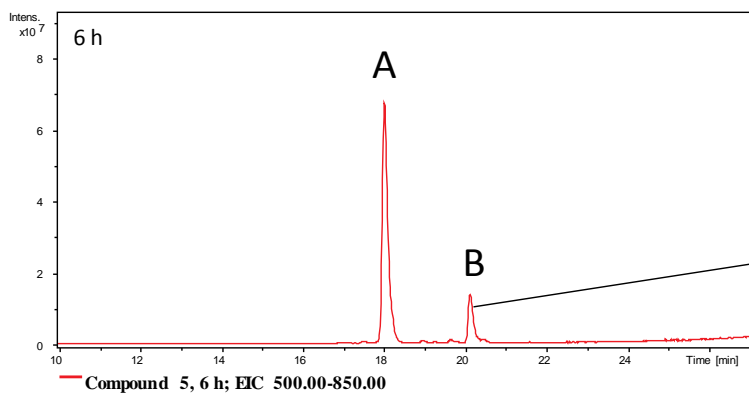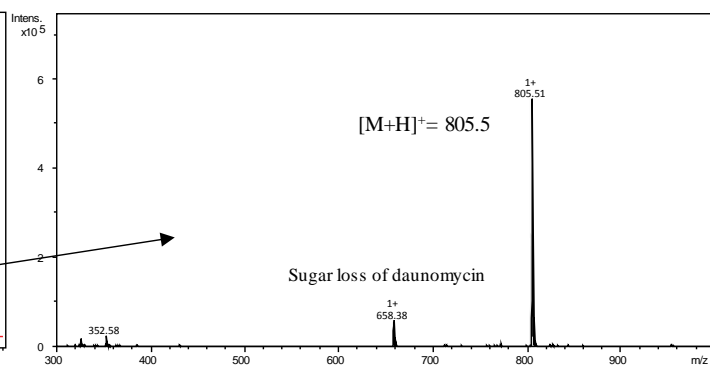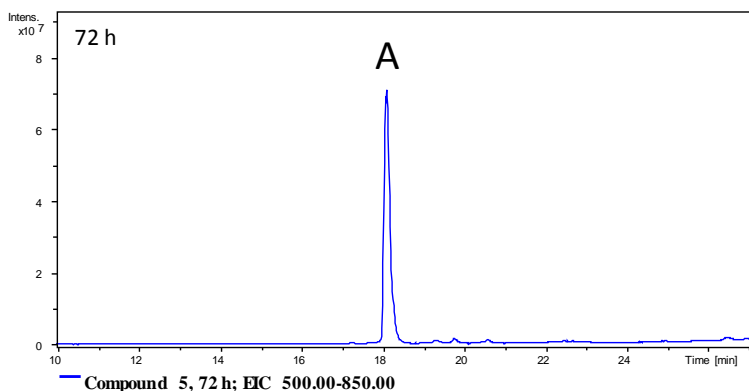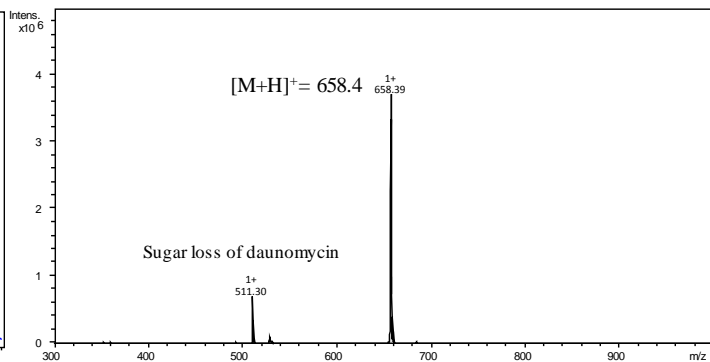

Supplement: S13 Fig — (PDF) [file pone.0178632.s013.pdf]

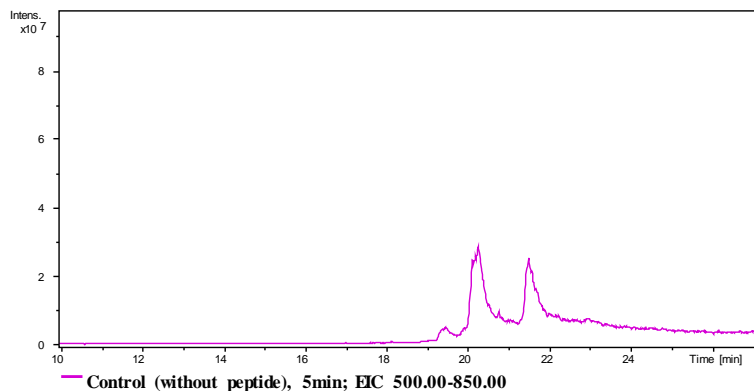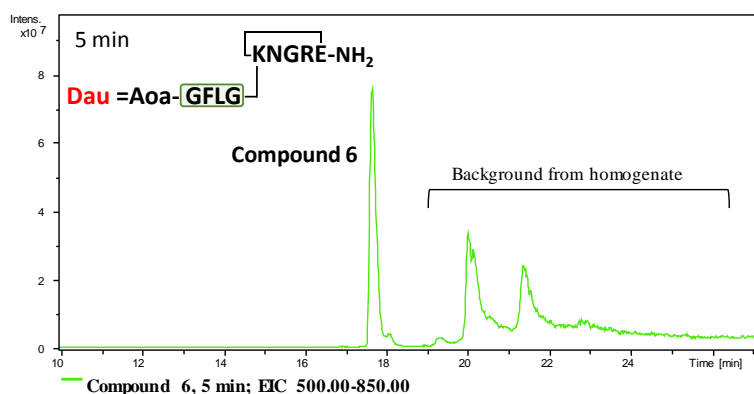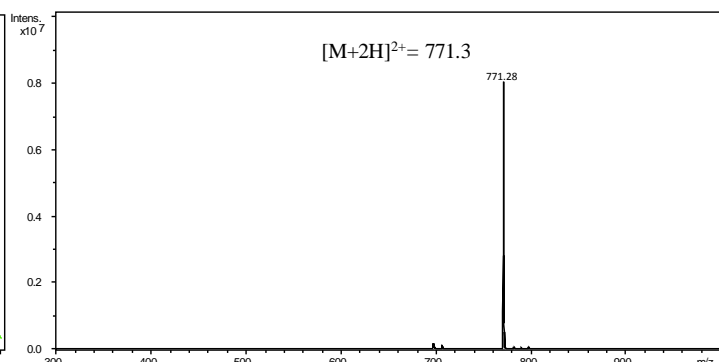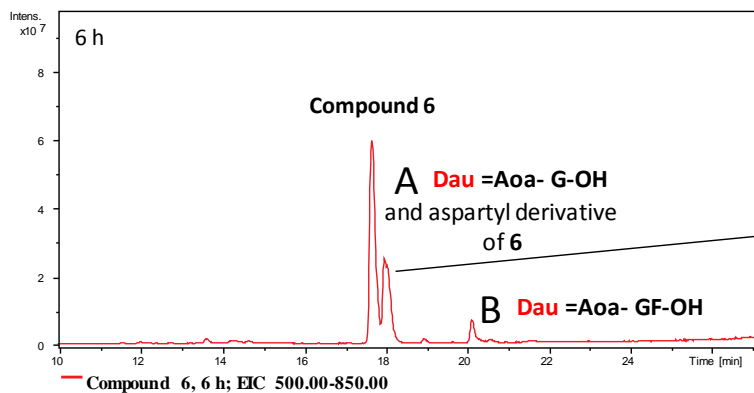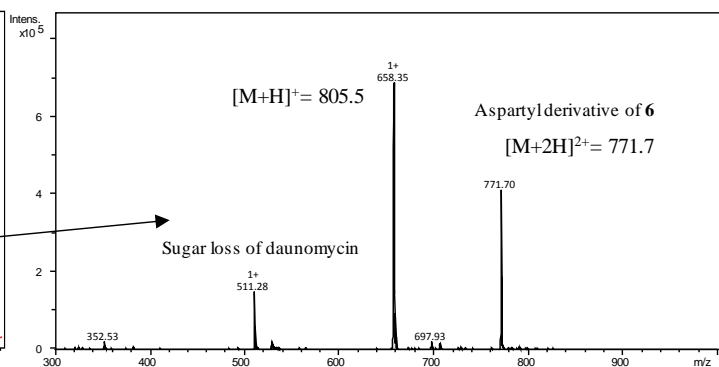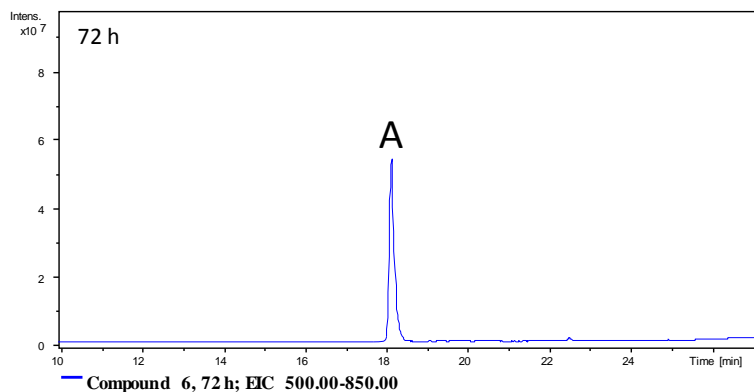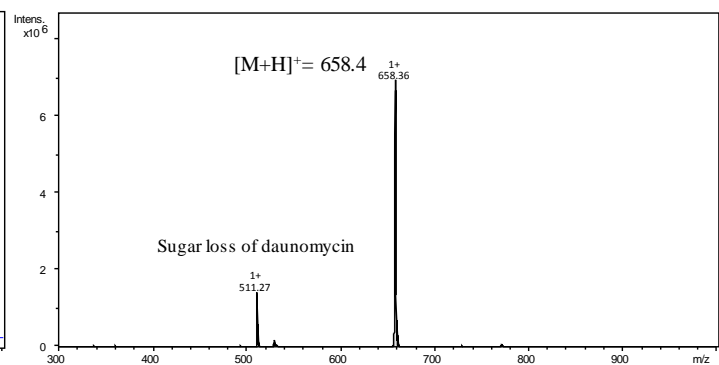

Supplement: S14 Fig — (PDF) [file pone.0178632.s014.pdf]

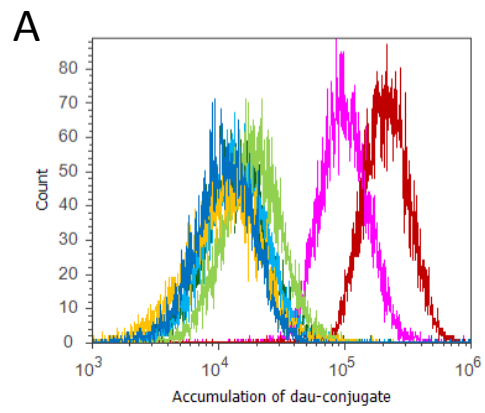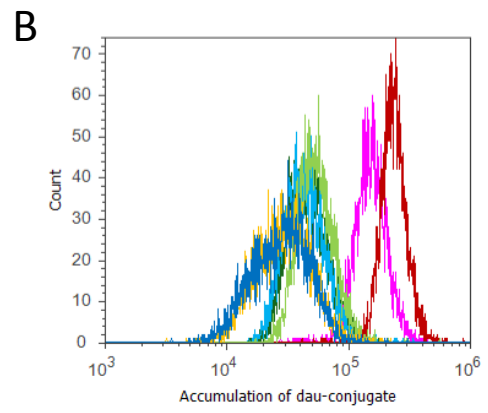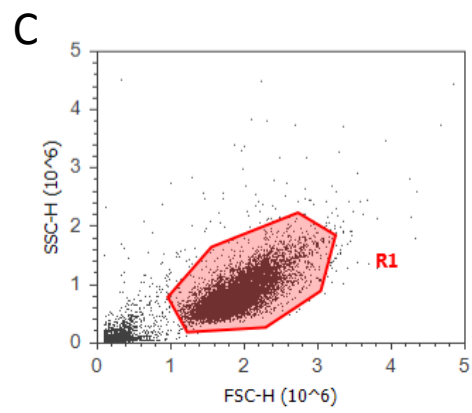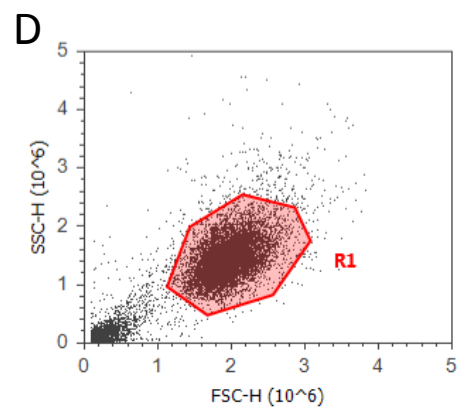

Supplement: S15 Fig — Accumulation of daunomycin-conjugates in (A) HT1080 and in (B) HT29 cells. Uptake of compound 1 (yellow); 2 (red); 3 (light green); 4 (dark green); 5 (purple); 6 (light blue). Empty control (the autofluorescence of the cells) is indicated with dark blue. Side scatter versus forward scatter values for (C) HT1080 and for (D) HT29 cells. (PDF) [file pone.0178632.s015.pdf]
